# Identifying species of moths (Lepidoptera) from Baihua Mountain, Beijing, China, using DNA barcodes

**DOI:** 10.1002/ece3.1110

**Published:** 2014-05-20

**Authors:** Xiao F Liu, Cong H Yang, Hui L Han, Robert D Ward, Ai-bing Zhang

**Affiliations:** 1 College of Life Sciences, Capital Normal UniversityBeijing, 100048, China; 2 School of Forestry, Experiment Center, Northeast Forestry UniversityHaerbin, 150040, China; 3 Wealth from Oceans Flagship, CSIRO Marine and Atmospheric ResearchGPO Box 1538, Hobart, Tasmania, 7001, Australia

**Keywords:** Bayesian, cytochrome c oxidase subunit I, diagnostic character, DNA barcode, genetic distance, Lepidoptera, maximum likelihood, moths, neighbor joining

## Abstract

DNA barcoding has become a promising means for the identification of organisms of all life-history stages. Currently, distance-based and tree-based methods are most widely used to define species boundaries and uncover cryptic species. However, there is no universal threshold of genetic distance values that can be used to distinguish taxonomic groups. Alternatively, DNA barcoding can deploy a “character-based” method, whereby species are identified through the discrete nucleotide substitutions. Our research focuses on the delimitation of moth species using DNA-barcoding methods. We analyzed 393 Lepidopteran specimens belonging to 80 morphologically recognized species with a standard cytochrome c oxidase subunit I (COI) sequencing approach, and deployed tree-based, distance-based, and diagnostic character-based methods to identify the taxa. The tree-based method divided the 393 specimens into 79 taxa (species), and the distance-based method divided them into 84 taxa (species). Although the diagnostic character-based method found only 39 so-identifiable species in the 80 species, with a reduction in sample size the accuracy rate substantially improved. For example, in the Arctiidae subset, all 12 species had diagnostics characteristics. Compared with traditional morphological method, molecular taxonomy performed well. All three methods enable the rapid delimitation of species, although they have different characteristics and different strengths. The tree-based and distance-based methods can be used for accurate species identification and biodiversity studies in large data sets, while the character-based method performs well in small data sets and can also be used as the foundation of species-specific biochips.

## Introduction

Biological taxonomy is essentially about the philosophy of relationships among organisms. Many kinds of relationships can be described, including those based on phenetics, cladistics, and patristics. Because there are so many perspectives, many different taxonomic methods and approaches have been proposed, leading to the development of different schools of thought. One fundamental requirement of taxonomy is precise and accurate species identification, and for this DNA barcoding has proven to be a powerful and effective new tool (Hebert et al. 2003a; le Gall and Saunders 2010), and can be integrated with alternative approaches to further improve the accuracy of identification.

DNA barcoding (http://www.barcodinglife.org) has gained widespread prominence during the past ten years as part of the worldwide campaign to develop a global biodiversity inventory (Ratnasingham and Hebert 2007; Zhang et al. 2010). On 10 April 2014, there were 2,971,941 barcode sequences from 211,654 species (Animals 143,771; Plants 52,514; Fungi & Other Life 15,369) in the Barcode of Life Database (BOLD) (http://www.barcodinglife.org). However, some reservations still remain about the utility of DNA barcodes, with two main issues, the choice of barcode gene and methods for species assignments, being of central concern (Dai et al. 2012).

The barcode gene was proposed to be the 5′ segment of the mitochondrial (mt) cytochrome c oxidase subunit I (COI) gene (648 bp) (Hebert et al. 2003b,c2003c). This has proved to be a great success in many animal groups (Hebert et al. 2004b) and has been selected as a the standard barcode gene for animal taxa (Kress and Erickson 2012). Examples include insects (Hajibabaei et al. 2006), birds (Tavares and Baker 2008), fishes (Ward et al. 2009) and crustaceans (Radulovici et al. 2009), and is also effective for algae (Saunders 2005). In practice, the choice of the barcode gene is still somewhat contentious. Although the COI gene can be used effectively for Lepidoptera and some other insects, many studies have employed other genes (COX2、18S、28S). COI is an ineffective species discriminator for plants and fungi, where other genes have been nominated as barcode markers, for example, *rbc*L and *mat*K for plants (Hollingsworth et al. 2009), and ITS for fungi (Seifert 2009).

The second hotly debated issue concerns the method used to assign a queried sequence to a particular species in the reference data base (Hebert et al. 2003b). The BOLD web site (http://www.barcodinglife.org) and data base are committed to this purpose. At present, a large variety of available approaches has been proposed. These include similarity (Little and Stevenson 2007), tree-based (Elias et al. 2007), distance-based (Bergmann et al. 2013), and diagnostic character methods (Hebert et al. 2003b; Bergmann et al. 2013). BOLD essentially uses a distance-based method (Ratnasingham and Hebert 2007). Decision-theoretic methods (Abdo and Golding 2007) can also be used, such as Bayesian (Munch et al. 2008a,b2008b), pure clustering (Austerlitz et al. 2009), BP-based (Zhang et al. 2008; Zhang and Savolainen 2009), and fuzzy-set-theory-based methods (Zhang et al. 2011). We chose to compare tree-based, distance-based, and diagnostic character methods in our project to identify moth species. Tree-based methods compare the evolutionary relationships between the unknown and reference sequences to determine species names; this is a commonly used DNA barcode method for species assignation. Distance-based methods use patristic distance between species or between populations within a species to identify specimens. It is measured by a variety of parameters. This method requires an assessment of the genetic distance within and between species, the former generally being appreciably smaller than the latter leading to the so-called “DNA barcoding gap” (Meyer and Paulay 2005). The existence of a DNA barcoding gap means that unknown sequences can be assigned to species. The diagnostic character method allows species identification through the presence or absence of discrete nucleotide substitutions (character states) within the DNA sequence (Rach et al. 2008). This approach has been used for rapid species identification in small samples of *Drosophila* (Yassin et al. 2010) and Odonata (Rach et al. 2008), using CAOS (Characteristic Attribute Organization System) software (Neil Sarkar et al. 2002).

Moths and butterflies constitute the large insect order Lepidoptera, one of the most widespread and widely recognizable orders in the world (Resh and Card 2003). The term was coined by Linnaeus in 1735 (Harper 2014). It is currently estimated to comprise 174,250 species, in 126 families and 46 super families (http://www.ucl.ac.uk/taxome/lepnos.html). Lepidoptera show many variations to the basic body structure that have evolved to adapt to multiple lifestyles and widespread geographic ranges. Recent estimates suggest that the order may have more species than earlier thought (Kristensen et al. 2007).

The large biological diversity of moths makes this group especially suitable for studying DNA barcode-based specimen identification. We sampled a total of 393 individuals comprising 80 moth species from the Baihua Mountain (near Beijing, China), located in the Baihua Mountain National Nature Reserve. This is a forest ecosystem nature reserve and the largest area of high insect and Lepidoptera biodiversity in the Beijing region.

## Materials and Methods

### Lepidoptera sampling

We used single 250-W universal incandescent lamp traps (OSRAM, Germany) powered by 220-V alternating current (AC) to sample moth communities during two summer periods, 3–5 July 2010 and 3 July 2011. The site was Baihua Mountain (latitude 39.85°, longitude 115.56°, elevation 733 m). In all, 393 individuals, comprising 80 moth species, were collected (Table 1 and Appendix S1). Specimens were frozen to facilitate curation and identification. Individuals were identified to species using available taxonomic keys, and specimens vouchered in the museum collection. Recognized taxonomic experts (Drs. Chunsheng Wu, Fuqiang Chen, Huilin Han, and Houshuai Wang) performed or verified determinations of common species. Trichoptera specimens, used as an outgroup, were identified by Lianfang Yang and preserved at Capital Normal University (Beijing).

**Table 1:** Taxonomic summary of specimens investigated

Taxon	Number of genera	Number of species	Number of specimens
Amatidae	1	1	4
[Table-fn tf1-1]Arctiidae	7	12	58
Bombycidae	1	1	2
Brahmaeidae	1	1	2
Cossidae	1	1	2
Crambidae	12	14	90
Lasiocampidae	1	1	2
Limacodidae	3	3	35
Lymantriidae	4	4	28
2Noctuidae	16	18	55
Notodontidae	6	6	26
Pyralidae	7	7	21
Sphingidae	9	10	66
Thyatiridae	1	1	2
3Trichoptera	1	1	3
4Total	70	80	393

1 This data used as a separate data set and named the Arctiidae data set.

2 This data used as a separate data set and named the Noctuidae data set.

3 Trichoptera is the outgroup.

4 Statistical results do not include the outgroup.

### DNA extraction, PCR, and sequencing

DNA samples were prepared from individual insects by extraction of total DNA from frozen animals or animals preserved in 95% ethanol(alcohol). Genomic DNA was extracted using the BIOMEDDN easy kit. The COI gene was amplified via PCR using rTaq (TAKARA) with the “universal” DNA primers of the mitochondrial cytochrome c oxidase subunit I gene (COI), LCO1490 (GGTCAACAAATCATAAAGATATTGG), and HCO2198 (TAAACTTCAGGGTGACCAAA AAATCA) (Vrijenhoek 1994).

The amplification reaction was performed in a total volume of 50 mL, including 2 × EasyTaq 25 mL, 1 mL of each primer (10 mmol/L), 0.5 mL of template DNA and 22.5 mL of distilled water. The PCR conditions for the COI gene were as follows: 94°C for 5 min, 30 cycles of 94°C for 30 sec, 53°C for 30 sec, 72°C for 30 sec, and a final extension at 72°C for 5 min. The PCR products were confirmed by 1.5% agarose gel electrophoresis and stained with ethidium bromide. Sequencing was performed with an ABI3130 (Applied Biosystems) automatic sequencer.

### Construction of phylogenetic trees

The raw DNA sequences were all checked manually by Chromas (Mccarthy 1996). After trimming the ends, they were aligned using MUSCLE (Edgar 2004) with default parameters. In order to analyze the effect of different data sizes on the success rate of specimen identification, we assembled three data sets: the full data set (393 individuals from 80 species), an Arctiidae data set (58 individuals from 12 species) and a Noctuidae data set (55 individuals from 18 species).

We constructed a neighbor-joining tree (N-J) (Saitou and Nei 1987) for each data set, with the Trichoptera as an outgroup taxon. N-J trees were built using MEGA 5.0 (Tamura et al. 2011) with a K2P molecular evolutionary model (Hebert et al. 2003b,c2003c). Branch supports were estimated using 1000 bootstrap replications. All other parameters used default settings. Successful identification was inferred when sequences from the same species formed a monophyletic group, although treating reciprocal monophyly as a measure of species identification success remains controversial (Rubinoff 2006).

We also constructed maximum likelihood and Bayesian phylogenies for the complete data set, and compared the topologies of the three phylogenies. The maximum likelihood tree (ML) were built using MEGA 5.0 (Tamura et al. 2011) with a K2P molecular evolutionary model. Branch supports were estimated using 1000 bootstrap replications. All other parameters used default settings. The Bayesian tree were built using BEAST 1.75 (Drummond and Rambaut 2007; Drummond et al. 2012) with a GTR molecular evolutionary model (Tavar 1986) and a Yule process for the tree prior (Gernhard 2008). Posterior estimates of parameters were obtained using Markov chain Monte Carlo sampling (MCMC). We drew samples at10,000,000 steps (length of chain). To check for convergence to the stationary distribution, we ran two independent Markov chains. Sufficient sampling was checked by inspecting the effective sample sizes of parameters, which were all greater than 200. Finally, the TreeAnnotator program assists in summarizing the information from a sample of trees produced by BEAST onto a single “target” tree.

### Species assignment and delimitation with distance-based methods

From previous studies, we know that DNA barcoding with COI is efficient when intraspecific diversity is lower than interspecific diversity, that is when sequences sampled within the same species are always more similar than sequences from different species. A large DNA barcoding gap means that species assignation of unknown sequences is quick and easy. Conversely, small or zero DNA barcoding gaps blur species boundaries, making it difficult or impossible to clearly assign specimens.

Distance-based methods of species allocation are capable of determining the statistical significance of species identification success rates. These include the best close match (BCM) (Meier et al. 2006) and the minimum distance (MD) method, utilizing “single-sequence-omission” or “leave-one out” simulations; these have a wide range of applications (Dai et al. 2012). The data set can be partitioned into candidate species through threshold distances. The General Mixed Yule Coalescent (GMYC) model (Pons et al. 2006; Monaghan et al. 2009) can be used to build groups (termed MOTUs, for Molecular Operational Taxonomic Units) (Floyd et al. 2002), as can methods based on Markov Chain Clustering and Automatic Barcode Gap Discovery (ABGD) (Puillandre et al. 2012). The ABGD approach is different from other methods as it does not require threshold distances [such as 3% divergence (Smith et al. 2005) or the 10 × rule (Hebert et al. 2004a)] to be set as it automatically finds the distance where the barcoding gap is located. This method proposes a standard definition of the barcode gap and can be used to partition the data set into candidate species even when two distributions overlap. We used the ABGD approach to analyze our data set.

We submitted fasta sequences from the three data sets (all 393 individuals, the 55 Arctiidae, and the 58 Noctuidae) to the ABGD online website (http://wwwabi.snv.jussieu.fr/public/abgd/abgdweb.html), with P (prior intraspe-cific divergence) set from 0.001 to 0.1 and Steps set to 10; X (minimum relative gap width) set to 1.5; Nb bins (for distance distribution) set to 20; we selected the Kimura (K80) model and set TS/TV to 2.0. The results were also compared with morphological data.

### The character-based method and diagnostic characters

At present, this method has been deployed in *Drosophila* (Yassin et al. 2010) and Odonata (Rach et al. 2008) using CAOS software (Neil Sarkar et al. 2002), which requires a guide tree to identify diagnostic characters. However, our approach does not use a tree to find those special nucleotide sites that can distinguish species, and is simpler in form and procedure. In our study, a diagnostic character is a single nucleotide that is diagnostic for that taxon; multiple or complex diagnostic nucleotide positions are not treated.

We identified the species diagnostic characters of 393 sequences (full data set), 55 sequences (Arctiidae), and 58 sequences (Noctuidae), then determined the number of species with diagnostic characters divided by the total number of species. This gives the success ratio of species diagnostic characters; an important index for testing the efficiency of the method. In addition, we also determined diagnostic characters at family level for the 393 sequences (full data set), then calculated the success ratio of family diagnostic characters. The computer program was developed by the Genetic Diversity and Evolution Group of Capital Normal University (http://smkxxy.cnu.edu.cn/szll/zrjs/js/4557.htm).

### Success rate of species identification and confidence intervals

The success rate of species identification is defined by the following formula (Zhang et al. 2008).


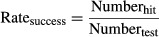
(1)

where Number_hit_ and Number_test_ are, respectively, the numbers of sequences successfully hit by the method under study and the number of total query sequences examined. A success hit is counted if a query is assigned to its correct species name in the data base.

Binary data indicating the presence (successful identification) or absence (failed identification) of a specific attribute are often modeled as random samples from a Bernoulli distribution with parameter *prob*, where *prob* is the proportion in the population with that attribute. A (1-a) level confidence interval (CI) for *prob* is calculated by the following formula (Dunlop 1999):


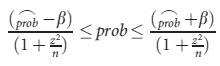
(2)

where *α* = 0.05, 
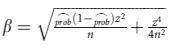
, *z* = 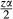


(*n* is the number of replications, and *z* is the critical value corresponding to an area 1-*α* under the standard normal curve).

## Results

### Phylogenetic inference and haplotype analysis

Three hundred and ninety-three specimens of Lepidoptera were obtained from Baihua Mountain (Appendix S1; see Materials and Methods for details). All individuals were successfully sequenced for the barcode portion of the COI gene, and all sequences were used in the subsequent alignment analysis. The resultant trimmed COI sequence had a length of 615 bp. All sequences have been deposited in GenBank with accession numbers KC4976-KC5063; JX392728-JX392799; KF704397-KF704654. In addition to the full data set, this research also investigated two smaller data sets: the families Arctiidae and Noctuidae (Table 1).

**Figure 1:**
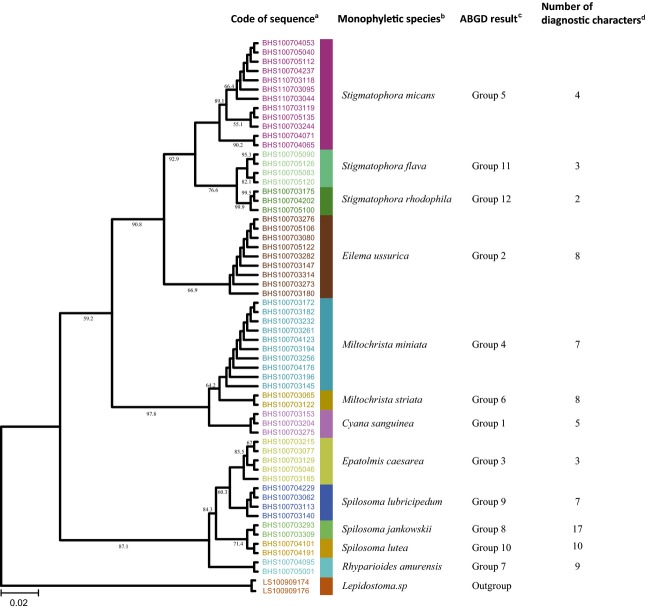
Phylogenetic trees (N-J) of 58 Arctiidae moth specimens. Clades with different colors indicate different species. Numbers above branches indicate bootstrap values (100 not shown). The data set consists of 12 Arctiidae species from 55 specimens. All sequences cluster into monophyletic groups and these monophyletic groups and morphological identifications are consistent. These groups were also devised by ABGD software and are consistent with morphological data. Every morphological species has 2–17 diagnostic characters. All methods permit excellent assignment of species. ^**a**^Code of sequence is the serial number of specimens, and different colors indicate different clustering relationships. ^**b**^Monophyletic species obtained by the Tree-based method (N-J tree). ^**c**^Groups obtained by the Distance-based method (ABGD Software). ^**d**^Numbers of diagnostic characters obtained by the Character-based method.

**Figure 2:**
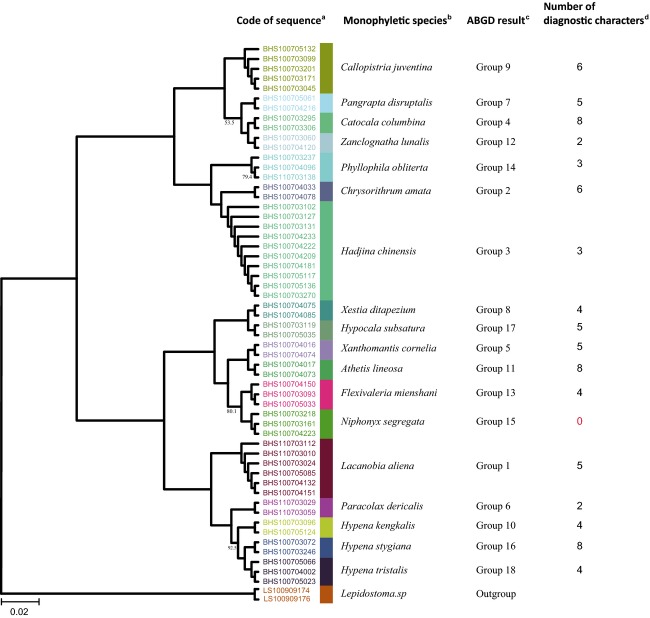
Phylogenetic trees (N-J) of 55 Noctuidae moth specimens. Clades with different colors indicate different species. Numbers above branches indicate bootstrap values (100 not shown).As shown in the figure, the data set consists of 18 Noctuidae species from 58 specimens. All sequences cluster into a monophyletic groups and morphological identification are consistent. These groups were also devised by ABGD software and are consistent with morphological data. Excepting *Niphonyx segregate* which did not have any diagnostic characters, every morphological species has 2–8 diagnostic characters. All methods permit excellent to very good assignment of species.^**a**^Code of sequence is these specimens serial number and different colors indicate different clustering relationship by N-J tree. ^**b**^these monophyletic species obtained by Tree-based method (N-J tree). ^**c**^these groups obtained by Distance-based method (ABGD Software). ^**d**^these diagnostic character obtained by Character-based method.

We obtained three N-J trees, one based on the full data set (Appendix S1), one on the Arctiidae (Fig. 1), and one on the Noctuidae (Fig. 2). In the full data set (393 individuals from 80 species), the Arctiidae (58 individuals from 12 species) and the Noctuidae (58 individuals from 12 species), the monophyletic ratio at the genus level is, respectively, 94.28% (64/70 genera), 85.71% (6/7 genera), and 100% (16/16 genera), and the monophyletic ratio at the species level is, respectively, 98.75% (79/80 species), 100% (12/12 species), and 100% (18/18 species) (Table 2, see Materials and Methods for details). In the full data set, the four genera *Callambulyx*, *Marumba*, *Spilosoma*, and *Miltochrista* and the species *Callambulyx tatarinovi* were not monophyletic; in the Noctuidae data set, the species *Niphonyx segregata* was not monophyletic. Apart from *Callambulyx tatarinovi,* all species assignments are consistent with the morphological data.

**Table 2:** Results of the three data sets analyzed using three DNA barcoding methods

Success ratio of different methods	Full data set (%)	Arctiidae data set (%)	Noctuidae data set (%)
1Monophyletic ratio at genus level	94.28	85.71	100
2Monophyletic ratio at species level	98.75	100	100
3Success ratio of ABGD group at species level	90.47	100	100
3Success ratio of diagnostic characters at family level	50.00	–	–
3Success ratio of diagnostic characters at species level	48.75	100	94.44

1 Tree-based method (Phylogenetic tree) by PAUP and AbouTree Software.

2 Distance-based method by ABGD Software.

3 Diagnostic character-based method by a computer program of searched diagnostic characters.

We also built the Maximum Likelihood (ML) tree and the Bayesian tree (BI) (Appendix S3). The monophyletic ratios at the species level for the ML tree and the BI tree are, respectively, 97.5% (82/80 species) and 98.75% (81/80 species). The species *Callambulyx tatarinovi* was non-monophyletic in all three approaches. While all three approaches give very similar success rates, there is a vast difference in the time taken: the N-J approach took only five minutes to built the tree while the ML and BI approaches took, respectively, about 20 and 30 h.

### Intraspecific and interspecific variation, and DNA barcoding gaps

In the full data set, there was an average interspecific K2P (Kimura two parameter model) (Kimura 1980) distance of 0.1368 ± 0.0212 which is about 30 times (29.73) larger than the mean intraspecific distance of 0.0046 ± 0.0127 (Table 3). In the Arctiidae and Noctuidae data sets, the multiples are, respectively, 55 (0.1091 ± 0.0216/0.0020 ± 0.0034 = 54.55) and 370 times (0.1109 ± 0.0158/0.0003 ± 0.0012 = 369.66) (Table 3).

**Table 3:** Intraspecific distance and Interspecific distance

Name	Mean	Range	SD^3^	SE^2^
Full data set intraspecific Distance	0.0046	0–0.0973	0.0127	3.1299e-04
Full data set interspecific Distance	0.1368	0.0164–0.2284	0.0212	7.7135e-05
Arctiidae data set of intraspecific Distance	0.0020	0–0.0182	0.0034	2.5521e-04
Arctiidae data set of interspecific Distance	0.1091	0.0436–0.1574	0.0216	5.6178e-04
Noctuidae data set of intraspecific Distance	0.0003	0–0.0082	0.0012	1.2965e-04
Noctuidae data set of Interspecific Distance	0.1109	0.06820–0.1549	0.0158	4.2446e-04

1 SD is standard deviation.

2 SE is standard error.

**Figure 3:**
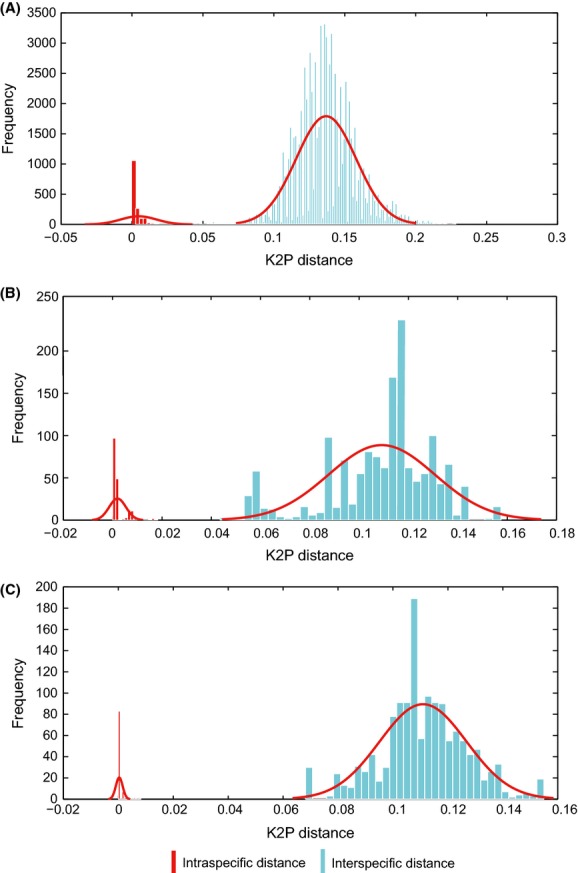
DNA barcoding gap analysis of COI gene sequence. (A) Full data set; (B) Arctiidae data set; (C) Noctuidae data set.

In the full data set, intraspecific distances range from 0 to 0.0973 and interspecific distance from 0.0164 to 0.2284. There is therefore no positive DNA barcoding gap (Fig. 3A). In the Arctiidae, intraspecific distances range from 0 to 0.0182 and interspecific distances range from 0.0436 to 0.1574; in the Noctuidae, intraspecific distances range from 0 to 0.0082 and interspecific distances range from 0.06820 to 0.1549 (Fig. 3B,C). Therefore, both the Arctiidae and Noctuidae data sets have positive DNA barcoding gaps.

### Species delimitation with the ABGD method

We submitted the sequences (fasta files) of all 393 individuals (full data set), 55 individuals (Arctiidae), and 58 individuals (Noctuidae) to the ABGD online website. One critical parameter of the ABGD method is the prior maximum divergence of intraspecific diversity (*P*). If this is set too high, the whole data set will be considered as a single species, and if set too low, only identical sequences will be considered as part of the same species (Puillandre et al. 2012). We set up values for the prior *P* ranging from 0.001 to 0.1.

ABGD outputs two partitions: the initial and recursive partitions. Generally, recursive partitions have more groups than initial partitions. However, recursive partitions are expected to better handle heterogeneities in the data set, while initial partitions are typically stable on a wider range of prior values and are usually close to the number of groups described by taxonomists (Puillandre et al. 2012).

**Figure 4:**
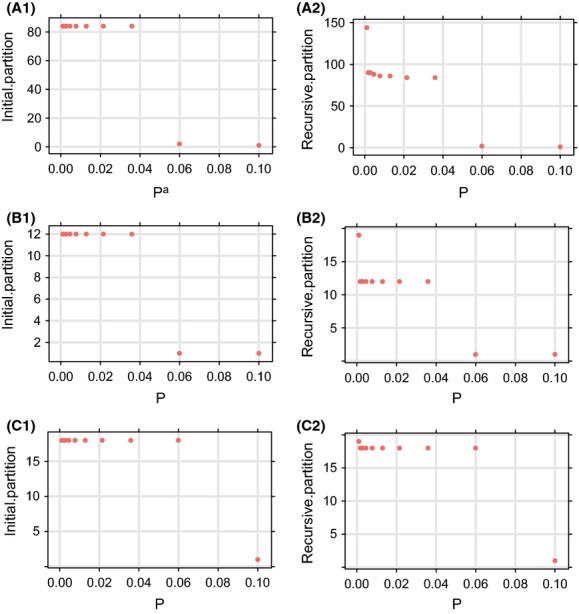
The automatic partition results by ABGD. (A1) Initial partition with the full data set; (B1) Initial partition with the Arctiidae data set; (C1) Initial partition with the Noctuidae data set; (A2) Recursive partition with the full data set; (B2) Recursive partition with the Arctiidae data set; (C2) Recursive partition with the Noctuidae data set; ^**a**^P is prior intraspecific divergence (*P*).

In the full data set (393 individuals, 80 species), results from initial partitions show that the number of groups ranges from 1 (when *P* = 0.1) to 84 (when *P* = 0.001), the latter corresponding to groups of identical sequence (Fig. 4A). The *P* value for the large range of 0.001–0.0359 gives 84 groups, close to the morphological data (Table 4). In the Arctiidae (58 individuals, 12 species) and Noctuidae (55 individuals, 18 species), initial partitions give 12 (for *P* = 0.001–0.0359) (Fig. 4B), and 18 (for *P* = 0.001–0.0599) groups, respectively (Fig. 4C), meaning that for both data sets this partition is fully consistent with the morphological data (Table 4).

**Table 4:** Result of partition by ABGD

	Number of groups of the full data set (*N*_g_)		Number of groups of the Arctiidae data set (*N*_g_)		Number of groups of the Noctuidae data set (*N*_g_)	
			
Prior intraspecific divergence (*P*)	Initial partition	Recursive partition	Initial partition	Recursive partition	Initial partition	Recursive partition
0.0010	84	144	12	19	18	19
0.0017	84	90	12	12	18	18
0.0028	84	90	12	12	18	18
0.0046	84	88	12	12	18	18
0.0077	84	86	12	12	18	18
0.0129	84	86	12	12	18	18
0.0215	84	84	12	12	18	18
0.0359	84	84	12	12	18	18
0.0599	2	2	–	1	18	18
0.1000	1	1	–	–	–	1

Number of species (*N*_s_) in the full data set is 80; Number of species (*N*_s_) of the Arctiidae data set is 12 and Number of species (*N*_s_) of the Noctuidae data set is 18.

In the full data set (80 species), recursive partitions show that the number of groups ranges from 1 (when *P* = 0.1) to 144 (when *P* = 0.001), the latter corresponding to groups of identical sequence (Fig. 4A). The ABGD method and some others predict species based on the DNA barcoding gap. However, in the full data set, there is some overlap between intraspecific and interspecific sequence variation and no positive DNA barcoding gap ( Fig.3A). Without a DNA barcoding gap, one cannot precisely estimate the number of groups. However, initial partitions with *P* values ranging from 0.0215 to 0.0359 (Table 4) give 84 groups. In this data set, the 80 species are partitioned into 84 groups (Table 4), with 6 groups not being consistent with the morphological data.

Both the Arctiidae and Noctuidae data sets have distinct DNA barcoding gaps. In the Arctiidae (12 species), result of recursive partitions show that the number of groups ranges from 1 (when *P* = 0.1) to 19 (when *P* = 0.001), the latter corresponding to groups of identical sequence (Fig. 4B). Using the range of the DNA barcoding gap (0.0182 to 0.0436, Table 3) to determine *p* values, then the number of groups is 12 (Table 4), fully consistent with the morphological data. In the Noctuidae (18 species), recursive partitions show that the number of groups ranges from 1 (when *P* = 0.1) to 19 (when *P* = 0.001), the latter corresponding to groups of identical sequence (Fig. 4C). Using the range of the DNA barcoding gap (0.0082 to 0.0682, Table 3) to determine *P* values gives 18 groups (Table 4), fully consistent with the morphological data.

### Diagnostic character states from molecular sequences

In the full data set (14 families, 80 species), we found 7 of the 14 families have diagnostic characters, giving a success ratio of 50%. Numbers of diagnostic characteristics range from 1 to 3, with the families Bombycidae, Lasiocampidae, Limacodidae, and Thyatiridae having only a single diagnostic character and the families Amatidae and Brahmaeidae having the most (three) (Table 5). Of the 80 species, 39 have diagnostic characteristics, a success ratio of 48.75%. Numbers of diagnostic characteristics range from 1 to 6, with 25 species having only one and *Sphrageidus similis* having six (Table 6).

**Table 5:** Character-based DNA barcodes for Moths of the BHS Mountain at family level

Family	Diagnostic characters	Number of characters	Number of specimens
Amatidae	T_28 T_30 G_191	3	4
Bombycidae	A_404	1	2
Brahmaeidae	G_363 C_364 G_512	3	2
Lasiocampidae	T_506	1	2
Limacodidae	G_384	1	32
Pyralidae	A_414 A_415	2	21
Thyatiridae	A_210	1	2

There were a total of fourteen families of Lepidoptera, and seven families have diagnostic characteristics (7/14, 50%).

**Table 6:** Character-based DNA barcodes for 39 species of moths from the BHS Mountain

Taxon	Family	Diagnostic characters	Number of characters	Number of individuals of the species
*Amata ganssuensis*	Amatidae	T_28 T_30 G_191	3	4
*Ambulyx ochracea*	Sphingidae	G_229 G_515	2	6
*Amorpha amurensis*	Sphingidae	G_275 G_500	2	2
*Brahmaea christophi*	Brahmaea	G_363 C_364 G_512	3	2
*Callopistria juventina*	Noctuidae	G_110	1	5
*Catocala columbina*	Noctuidae	G_236	1	2
*Chrysorithrum amata*	Noctuidae	C_515	1	2
*Cnaphalocrocis medinalis*	Crambidae	T_37	1	2
*Dendrolimus tabulaeformis*	Lasiocampidae	T_506	1	2
*Eilema ussurica*	Arctiidae	C_545	1	9
*Eoophyla sinensis*	Crambidae	G_131 A_438	2	2
*Epatolmis caesarea*	Arctiidae	G_421	1	5
*Flexivaleria mienshani*	Noctuidae	G_5	1	3
*Gluphisia crenatameridionalis*	Notodontidae	G_249 A_444	2	4
*Hypena kengkalis*	Noctuidae	G_371	1	2
*Hypena stygiana*	Noctuidae	C_26 C_41	2	2
*Hypena tristalis*	Noctuidae	G_431	1	3
*Kuromondokuga niphonis*	Lymantriidae	T_41 C_317 C_503	3	2
*Lamprosema commixta*	Crambidae	G_356	1	9
*Lophocosma nigrilinea*	Notodontidae	C_14 C_137 G_335	3	2
*Micromelalopha sieversi*	Notodontidae	G_17	1	11
*Narosoideus flavidorsalis*	Limacodidae	A_386	1	17
*Nerice hoenei*	Notodontidae	C_77 G_233 G_257 C_259	4	3
*Pangrapta disruptalis*	Noctuidae	G_95 C_203	2	2
*Parasa consocia*	Limacodidae	G_326 C_335	2	12
*Rhagastis mongolianamongoliana*	Sphingidae	C_248	1	2
*Sphinx ligustriconstricta*	Sphingidae	G_254	1	2
*Sphrageidus similis*	Lymantriidae	A_46 T_61 A_72 A_252 G_301 A_336	6	5
*Spilosoma lutea*	Arctiidae	C_509	1	2
*Stauropus basalis*	Notodontidae	A_162	1	4
*Stenia charonialis*	Crambidae	T_265	1	3
*Stigmatophora rhodophila*	Arctiidae	C_254	1	3
*Stilpnotia candida*	Lymantriidae	G_329 G_533	2	2
*Teia parallela*	Lymantriidae	C_155	1	19
*Teliphasa elegans*	Pyralidae	C_401	1	3
*Termioptycha nigrescens*	Pyralidae	T_228	1	4
*Tethea albicostata*	Thyatiridae	A_210	1	2
*Theophila mandarina*	Bombycidae	A_404	1	2
*Xanthomantis cornelia*	Noctuidae	C_266	1	2

There were a total of eighty species of Lepidoptera and thirty-nine have diagnostic characteristics (39/80, 48.75%).

In the Arctiidae (58 sequences, 12 species) and Noctuidae (55 sequences, 18 species), we found 12 and 17 species, respectively, with diagnostic characteristics, giving success ratios of 100% and 94.44%. In the Arctiidae, numbers of diagnostic characteristics range from 2 to 17, with *Stigmatophora rhodophila* having the smallest number and *Spilosoma jankowskii* the most (Table 7). In the Noctuidae, numbers of diagnostic characteristics range from 2 to 8, with *Paracolax derivalis* and *Zanclognatha lunalis* have the smallest number and *Athetis lineosa, Catocala columbina,* and *Hypena stygiana* the most (Table 8).

**Table 7:** Character-based DNA barcodes for 12 Arctiidae species

Species	Diagnostic characters	Number of characters	Number of individuals of the species
*Cyana sanguinea*	C_6 G_74 T_236 G_242 T_512	5	3
*Eilema ussurica*	C_224 C_260 C_284 C_410 T_430 C_543 C_545 C_548	8	9
*Epatolmis caesarea*	C_101 G_421 T_539	3	5
*Miltochrista miniata*	T_26 T_35 C_159 T_161 A_383 G_413 C_473	7	10
*Miltochrista striata*	C_44 A_86 T_128 A_264 A_446 C_470 G_530 C_533	8	2
*Rhyparioides amurensis*	T_14 A_152 G_320 A_426 G_440 T_486 A_488 C_554 C_569	9	2
*Spilosoma jankowskii*	G_23 G_32 C_53 G_128 C_225 G_266 T_275 G_281 A_302 C_308 G_317 C_344 C_404 C_407 C_476 A_503 G_566	17	2
*Spilosoma lubricipedum*	C_35 A_347 G_359 C_446 C_488 T_578 C_599	7	4
*Spilosoma lutea*	C_89 C_149 G_200 C_293 C_320 C_365 C_389 C_434 C_485 C_509	10	2
*Stigmatophora flava*	C_83 G_212 C_375	3	4
*Stigmatophora micans*	C_2 C_455 A_521 C_590	4	12
*Stigmatophora rhodophila*	C_254 C_431	2	3

There were a total of twelve species of Arctiidae and all twelve have diagnostic characteristics (12/12, 100%).

**Table 8:** Character-based DNA barcodes for 17 Noctuidae species

Species	Diagnostic characters	Number of characters	Number of individuals of the species
*Athetis lineosa*	C_21 C_269 C_326 A_338 C_354 T_356 C_455 T_575	8	2
*Callopistria juventina*	G_110 G_200 T_317 G_362 G_380 C_551	6	5
*Catocala columbina*	C_35 C_74 C_89 C_197 G_236 T_323 C_491 C_560	8	2
*Chrysorithrum amata*	T_294 A_429 T_430 C_515 C_543 T_545	6	2
*Flexivaleria mienshani*	G_5 C_71 C_173 C_557	4	3
*Hadjina chinensis*	C_80 G_221 C_536	3	10
*Hypena kengkalis*	T_131 G_371 G_464 C_497	4	2
*Hypena stygiana*	C_26 C_41 G_56 G_164 C_392 G_401 C_488 C_542	8	2
*Hypena tristalis*	G_107 G_431 C_470 T_497	4	3
*Hypocala subsatura*	A_147 C_194 C_332 T_380 T_449	5	2
*Lacanobia aliena*	G_14 C_65 T_128 T_377 C_473	5	6
*Pangrapta disruptalis*	G_95 C_203 T_266 T_416 T_428	5	2
*Paracolax derivalis*	T_281 C_434	2	2
*Phyllophila obliterata*	A_230 C_272 G_509	3	3
*Xanthomantis cornelia*	A_33 C_179 C_266 C_350 C_554	5	2
*Xestia ditrapezium*	C_38 G_128 C_407 G_440	4	2
*Zanclognatha lunalis*	T_35 C_600	2	2

There were a total of eighteen species of Noctuiidae and seventeen have diagnostic characteristics (17/18, 94.44%).

## Discussions

DNA barcoding is an applied science, and can be used to quickly and accurately identify most animal species. However, biological systems are highly complex and barcoding is not fool proof. For example, for hybridizing species or very closely related sibling taxa, species separation may not be possible with the most commonly used barcoding gene, COI. Several approaches are available for assigning species names from a consideration of barcode sequences, including tree-based, distance-based, and character-based methods. Here, we compared these different approaches to see how they vary in their characteristics and effectiveness for species identification, using as a test example a collection of moths taken from light traps on the Baihua Mountain, China.

Many barcoding studies use phylogenetic trees to assign species names. The most commonly used tree approach is based on neighbor joining (this is not a true phylogenetic tree as it is based on phenetic distance). It does not depend on a barcoding gap (Wiemers and Fiedler 2007) and shows specimen relationships with evolutionary information; it is therefore expected to be quite reliable. We found that three different methods of tree construction – neighbor joining, maximum likelihood, and Bayesian inference – produced phylogenies with minimal differences in topology and accuracies of species assignation. All had species success rates for the full data set (393 individuals, 80 species) of about 98–99%. There was, however, a significant discrepancy in the computational efficiency of the three methods, with neighbor joining being the most rapid (∼ 5 min) and maximum likelihood and Bayesian inference much slower (∼ 20 and 30 h, respectively). Since an important goal of DNA barcoding is the rapid identification of species, and neighbor joining is so much faster than other methods, we believe that the neighbor-joining approach is very suitable for tree building in DNA barcoding.

Distance and character-based methods have some significant limitations. The distance-based method is very efficient when intraspecific diversity is lower than interspecific diversity (Puillandre et al. 2012), giving a barcode gap, but is much less effective where there is inconspicuous barcode gap. Without a DNA barcode gap, species boundaries will not be clear and intraspecific and interspecific taxonomic relationships may be confused as a consequence. In our full data set, the COI interspecific distance (0.1368 ± 0.0212%) is c. 30 times larger than intraspecific distance (0.0046 ± 0.0127), but there was no overall barcode gap. In the subsets of Arctiidae (58 individuals, 12 species) and Noctuidae (55 individuals, 18 species), interspecific/intraspecific ratios are c. 55 and 50, respectively, and both have positive barcoding gaps. Several attempts have been made to establish a standard threshold value between intraspecific and interspecific divergence [3% of divergence (Smith et al. 2005) or the 10 times rule (Hebert et al. 2004a)], but none can be generalized to all groups of organisms (Hebert et al. 2003b). There is no, and can be no, universal threshold of genetic distance that can be used to distinguish all species: some species pairs cannot be separated by COI sequences (Ward 2009).

In our distance-based analysis, we applied a method, Automatic Barcode Gap Discovery (ABGD), that automatically finds the distance where the barcode gap is located. This method proposes a standard definition of the barcode gap and can be used even when the two distributions overlap to partition the data set into candidate species (Puillandre et al. 2012). This approach greatly reduces the interference of artificial factors. In our full data set, the 80 species are partitioned into 84 groups (Table 4), with 6 groups not being consistent with the morphological data. It is possible that ABGD mistook subspecies for species. In any case, and despite the absence of a barcode gap, the ABGD method performed well for our full data set. It performed perfectly for the two family data subsets (which both had barcode gaps). We conclude that this approach is very effective for species classification.

The third identification method we used is the character-based approach, whereby species are identified through the presence or absence of discrete nucleotide substitutions (character states) within the DNA sequence (Rach et al. 2008). This approach has been deployed in *Drosophila* (Yassin et al. 2010) and Odonata (Rach et al. 2008), using CAOS (Characteristic Attribute Organization System) software (Neil Sarkar et al. 2002). However, the CAOS method requires a guide tree to identify diagnostic characters, whereas the approach we used is tree-free and simpler in form and procedure. We considered only individual nucleotides as characters; the use of multiple nucleotides as character states was not modeled nor studied.

One problem with character-based determination is that the larger the intraspecific sample size, the more rare variants are uncovered, and the number of diagnostic characters diminishes. However, a greater issue concerns numbers of species. We used data sets with 12 (Arctiidae), 18 (Noctuidae), and 80 species (full data set), finding, respectively, that 12/12 (100%), 17/18 (94.44%), and 39/80 (48.75%) species had diagnostic characteristics (Tables 8). So, as the number of species increases, the success ratio of diagnostic characters at the species level fell from 100% to 48% (Table 2). The character-based approach performs well on small samples, where it can yield quick and easy species identification, but less well on large samples. This conclusion was supported by a random selection of 12 species from the complete data set of 80 species: a success ratio of 12/12 (100%) was found.

We conclude that for large DNA barcode data sets, such as those arising from biodiversity assessments in natural ecosystems, tree-based and distance-based approaches to species identification perform better than character-based methods. Of several tree-based approaches, neighbor-joining works as well as maximum likelihood and Bayesian methods and is much faster to apply. We found that the ABGD distance method is also very effective, but the character-based approach performs poorly. However, in small data sets, the character-based approach is very effective and hence can be used, for example, to develop biochips for rapid species identification. Biochips enable researchers to quickly screen large numbers of biological analyses for a variety of purposes (Yang et al. 2014), and the character-based method can thus play an important role in pest control, customs, and quarantine.

As a kind of applied science, DNA barcode can quickly and accurately identify species. Biological systems are so complex that there is not a way to solve all problems. So the scientists have developed a variety of methods used to achieve this goal of quickly and accurately identify species. Comparing these methods provides a clear example of how they vary in their characteristics and effectiveness and how they can be applied to a complex biological system. Tree-based and distance-based methods can be used for accurate species identification in biodiversity studies. For example, these methods could be used to survey biodiversity and would facilitate species identification and would help to uncover new and cryptic species. The character-based method, while perhaps not as useful for species identification in large data sets, could be used to develop biochips for rapid identification of species in the small data sets. It plays an important role in pest control, customs, and quarantine. These are all important components of DNA barcoding work.

Three methods are used in this study to assign COI barcode sequences to species. Two of these methods, the distance-based methods using the DNA barcode gap and the diagnostic character (character-based) method, do not require tree construction. However, many studies to determine species by DNA barcoding utilize phylogenetic trees. The neighbor-joining tree approach does not depend on a barcoding gap (Wiemers and Fiedler 2007) and shows phylogenetic relationships with evolutionary information; it is therefore expected to be the most reliable. When used in the analyses of our data set, the three methods of phylogenetic inference – neighbor joining, maximum likelihood, and Bayesian methods – were found to produce phylogenies with minimal difference in topology. There is, however, a discrepancy in the computational efficiency of the three methods, with neighbor joining being the most rapid (∼ 5 min) and Bayesian inference being the slowest (∼ 30 h). Since the main goal of DNA barcoding is the rapid identification of spec-ies and NJ approach is faster than other methods, the NJ approach is best candidate to build tree in DNA barcoding.

Distance and character-based methods might therefore be preferable, but these two methods have significant limitations. The distance-based method is very efficient when intraspecific diversity for the COI gene is lower than interspecific diversity, giving a barcode gap, but is much less effective where there is inconspicuous barcode gap. DNA barcode gap is crucial in ensuring the effectiveness of distance-based method. If the DNA barcode gap is not prominent, the species boundaries will not be clear. It is possible that intraspecific and interspecific taxonomic relationships are confused as a consequence. On the other hand, the success rate of the character-based method is largely limited by the sample size of the data set. With increasing data quantity, both in numbers of species and numbers of specimens per species, success rates decline. So a comparison of the different approaches methods is important.

DNA barcoding for species assignation is efficient when intraspecific diversity is lower than interspecific diversity (Puillandre et al. 2012), that is when COI sequences sampled within the same species are always more similar than sequences sampled from different species. Then, DNA barcodes can be used as a highly effective identification tool, shortcutting the difficulties of morphologically based identification (Hebert et al. 2003b). However, when there is no positive DNA barcoding gap, there is difficulty in distinguishing sibling species. In our full data set (393 individuals, 80 species), the COI interspecific distance (0.1368 ± 0.0212%) is 30 times (29.73) larger than intraspecific distance (0.0046 ± 0.0127). Both the Arctiidae and Noctuidae data sets presented higher interspecific variation (0.1091 ± 0.0216 for Arctiidae, 0.1109 ± 0.0158 for Noctuidae) than intraspecific variation (0.0020 ±0.0034 for Arctiidae, 0.0003 ± 0.0012 for Noctuida). The interspecific/intraspecific ratios are about 55 and 50 for Arctiidae and Noctuidae, respectively. These values are thus fully consistent with the “10 times rule” of DNA barcoding (Hebert et al. 2003b), which suggests that sequ-ences divergent by more than 10X the mean intraspecific variation should be considered as distinct species. Intraspecific and interspecific distances did overlap slightly in the full data set (not in the Arctiidae nor Noctuidae data sets), but not enough to influence the efficiency of species identification. Sequences in the N-J phylogenetic tree form monophyletic groups and species identification is effective.

Although several attempts have been made to establish a standard threshold value between intraspecific and interspecific divergence [3% of divergence(Smith et al. 2005) or the 10 times rule(Hebert et al. 2004a)], none can be generalized to all groups of organisms (Hebert et al. 2003b). Even worse, and as highlighted in several studies, intra- and interspecific distances frequently overlap, and visually defining a threshold becomes difficult or impossible (Hebert et al. 2003b). We applied a method that automatically finds the distance where the barcode gap is located, called Automatic Barcode Gap Discovery (ABGD). This method proposes a standard definition of the barcode gap and can be used even when the two distributions overlap to partition the data set into candidate species(Puillandre et al. 2012). This distance-based method greatly reduces the interference of artificial factors. In the full data set, the 80 species are partitioned into 84 groups, with 6 groups not being consistent with the morphological data. For this reason, it is difficult to determine, and it is possible that ABGD mistook subspecies for species. As species level identifiers, barcode differences appear to accumulate quickly, making it possible to distinguish all but the youngest of sister species (Hebert and Gregory 2005). This is one of the largest possible method. In any case, we conclude that the ABGD method is effective for species classification.

However, a universal threshold of COI genetic distance values to distinguish taxonomic groups does not, and cannot, exist. Some pairs of species cannot be separated by the COI barcode sequence (Ward 2009). An alternative DNA barcoding approach is “character-based”, whereby species are identified through the presence or absence of discrete nucleotide substitutions (character states) within the DNA sequence (Rach et al. 2008). This approach has been deployed in *Drosophila* (Yassin et al. 2010) and Odonata (Rach et al. 2008), using CAOS (Characteristic Attribute Organization System) software (Neil Sarkar et al. 2002). However, the CAOS method requires a guide tree to identify diagnostic characters, and the approach we used is tree-free and is simpler in form and procedure.

One problem with character-based determination is that the larger the intraspecific sample size, the fewer diagnostic characters are found as rare variants are uncovered. However, the greatest issue concerns the numbers of species in the data set. We used data sets with 12, 18, and 80 species and we found, respectively, that 12/12 (100%), 17/18 (94.44%), and 39/80 (48.75%) species had diagnostic characteristics (Table 8). Thus, as the number of species increases, the “success ratio” of diagnostic characters of species level fell from 100% to 48% (Table 2). This approach performs better on small samples where it can yield quick and easy species identification. We randomly selected 12 species from the complete data set of 80 species and found a success rate of 12/12 (100%). This result confirms the above conclusion. Note that, as mentioned earlier, we here consider individual nucleotides as characters; the use of multiple nucleotides as character states was not modeled nor studied.

The character-based method, while perhaps not as useful for species identification in large data sets, could be used to develop biochips for rapid identification of species in the small data sets. Biochips enable researchers to quickly screen large numbers of biological analyses for a variety of purposes and the diagnostic character information may be better to design a probe of biochips (Yang et al. 2014). And thus the character-based method play an important role in pest control, customs, and quarantine.

## Supporting Information

**Appendix S1.** Taxon information, detail sampling sites, genes used.

**Appendix S2.** Phylogenetic trees (N-J) of 393 moth specimens constructed with the barcode sequence from the COI gene. Clades with different colors indicate different species.

**Appendix S3.** Maximum Likelihood tree and Bayesian tree.
