# Supplementary material for: Identifying species of moths (Lepidoptera) from Baihua Mountain, Beijing, China, using DNA barcodes
Source: Ecol Evol. 2014 May 20;4(12):2472–87. doi: 10.1002/ece3.1110 (PMC4203292; doi:10.1002/ece3.1110)
Supplement: Supplementary file 1 — Appendix S1. Taxon information, detail sampling sites, genes used. [file ece30004-2472-sd1.pdf]

Appendix SI. Sample ID on BHS mountain in China

Family	Species	Sample ID	Sequence	Sample locality	Coordinates °N	Coordinates °E
Amatidae	*Amata ganssuensi* (Grum-Grshimailo, 1890)	BHS100703.067	COI	BHS Mountain, Beijing	39.51	115.33
Amatidae	*Amata ganssuensi* (Grum-Grshimailo, 1890)	BHS100703.076	COI	BHS Mountain, Beijing	39.51	115.33
Amatidae	*Amata ganssuensi* (Grum-Grshimailo, 1890)	BHS100703.083	COI	BHS Mountain, Beijing	39.51	115.33
Amatidae	*Amata ganssuensi* (Grum-Grshimailo, 1890)	BHS100704.135	COI	BHS Mountain, Beijing	39.51	115.33
Arctiidae	*Cyana sanguinea* (Bremer et Grey, 1852)	BHS100703.153	COI	BHS Mountain, Beijing	39.51	115.33
Arctiidae	*Cyana sanguinea* (Bremer et Grey, 1852)	BHS100703.204	COI	BHS Mountain, Beijing	39.51	115.33
Arctiidae	*Cyana sanguinea* (Bremer et Grey, 1852)	BHS100703.275	COI	BHS Mountain, Beijing	39.51	115.33
Arctiidae	*Eilema ussurica* (Daniel, 1954)	BHS100703.080	COI	BHS Mountain, Beijing	39.51	115.33
Arctiidae	*Eilema ussurica* (Daniel, 1954)	BHS100703.147	COI	BHS Mountain, Beijing	39.51	115.33
Arctiidae	*Eilema ussurica* (Daniel, 1954)	BHS100703.180	COI	BHS Mountain, Beijing	39.51	115.33
Arctiidae	*Eilema ussurica* (Daniel, 1954)	BHS100703.273	COI	BHS Mountain, Beijing	39.51	115.33
Arctiidae	*Eilema ussurica* (Daniel, 1954)	BHS100703.276	COI	BHS Mountain, Beijing	39.51	115.33
Arctiidae	*Eilema ussurica* (Daniel, 1954)	BHS100703.282	COI	BHS Mountain, Beijing	39.51	115.33
Arctiidae	*Eilema ussurica* (Daniel, 1954)	BHS100703.314	COI	BHS Mountain, Beijing	39.51	115.33
Arctiidae	*Eilema ussurica* (Daniel, 1954)	BHS100705.106	COI	BHS Mountain, Beijing	39.51	115.33
Arctiidae	*Eilema ussurica* (Daniel, 1954)	BHS100705.122	COI	BHS Mountain, Beijing	39.51	115.33
Arctiidae	*Epatolmis caesarea* (Goeze, 1781)	BHS100703.077	COI	BHS Mountain, Beijing	39.51	115.33
Arctiidae	*Epatolmis caesarea* (Goeze, 1781)	BHS100703.129	COI	BHS Mountain, Beijing	39.51	115.33
Arctiidae	*Epatolmis caesarea* (Goeze, 1781)	BHS100703.185	COI	BHS Mountain, Beijing	39.51	115.33
Arctiidae	*Epatolmis caesarea* (Goeze, 1781)	BHS100703.215	COI	BHS Mountain, Beijing	39.51	115.33
Arctiidae	*Epatolmis caesarea* (Goeze, 1781)	BHS100705.046	COI	BHS Mountain, Beijing	39.51	115.33
Arctiidae	*Miltochrista miniata* (Forster)	BHS100703.145	COI	BHS Mountain, Beijing	39.51	115.33
Arctiidae	*Miltochrista miniata* (Forster)	BHS100703.172	COI	BHS Mountain, Beijing	39.51	115.33
Arctiidae	*Miltochrista miniata* (Forster)	BHS100703.182	COI	BHS Mountain, Beijing	39.51	115.33
Arctiidae	*Miltochrista miniata* (Forster)	BHS100703.194	COI	BHS Mountain, Beijing	39.51	115.33
Arctiidae	*Miltochrista miniata* (Forster)	BHS100703.196	COI	BHS Mountain, Beijing	39.51	115.33
Arctiidae	*Miltochrista miniata* (Forster)	BHS100703.232	COI	BHS Mountain, Beijing	39.51	115.33
Arctiidae	*Miltochrista miniata* (Forster)	BHS100703.256	COI	BHS Mountain, Beijing	39.51	115.33
Arctiidae	*Miltochrista miniata* (Forster)	BHS100703.261	COI	BHS Mountain, Beijing	39.51	115.33
Arctiidae	*Miltochrista miniata* (Forster)	BHS100704.123	COI	BHS Mountain, Beijing	39.51	115.33
Arctiidae	*Miltochrista miniata* (Forster)	BHS100704.176	COI	BHS Mountain, Beijing	39.51	115.33
Arctiidae	*Miltochrista striata* (Bremer et Grey, 1851)	BHS100703.065	COI	BHS Mountain, Beijing	39.51	115.33
Arctiidae	*Miltochrista striata* (Bremer et Grey, 1851)	BHS100703.122	COI	BHS Mountain, Beijing	39.51	115.33
Arctiidae	*Rhyparioides amurensis* (Bremer, 1861)	BHS100704.095	COI	BHS Mountain, Beijing	39.51	115.33
Arctiidae	*Rhyparioides amurensis* (Bremer, 1861)	BHS100705.001	COI	BHS Mountain, Beijing	39.51	115.33
Arctiidae	*Spilosoma jankowskii* (Oberthür, 1880)	BHS100703.293	COI	BHS Mountain, Beijing	39.51	115.33
Arctiidae	*Spilosoma jankowskii* (Oberthür, 1880)	BHS100703.309	COI	BHS Mountain, Beijing	39.51	115.33
Arctiidae	*Spilosoma lubricipedum* (Linnaeus, 1758)	BHS100703.062	COI	BHS Mountain, Beijing	39.51	115.33
Arctiidae	*Spilosoma lubricipedum* (Linnaeus, 1758)	BHS100703.113	COI	BHS Mountain, Beijing	39.51	115.33
Arctiidae	*Spilosoma lubricipedum* (Linnaeus, 1758)	BHS100703.140	COI	BHS Mountain, Beijing	39.51	115.33
Arctiidae	*Spilosoma lubricipedum* (Linnaeus, 1758)	BHS100704.229	COI	BHS Mountain, Beijing	39.51	115.33
Arctiidae	*Spilosoma lutea* (Hüfnagel, 1766)	BHS100704.101	COI	BHS Mountain, Beijing	39.51	115.33
Arctiidae	*Spilosoma lutea* (Hüfnagel, 1766)	BHS100704.191	COI	BHS Mountain, Beijing	39.51	115.33
Arctiidae	*Stigmatophora flava* (Motschulsky)	BHS100705.083	COI	BHS Mountain, Beijing	39.51	115.33
Arctiidae	*Stigmatophora flava* (Motschulsky)	BHS100705.090	COI	BHS Mountain, Beijing	39.51	115.33
Arctiidae	*Stigmatophora flava* (Motschulsky)	BHS100705.120	COI	BHS Mountain, Beijing	39.51	115.33
Arctiidae	*Stigmatophora flava* (Motschulsky)	BHS100705.126	COI	BHS Mountain, Beijing	39.51	115.33
Arctiidae	*Stigmatophora micans* (Bremer et Grey)	BHS110703.095	COI	BHS Mountain, Beijing	39.51	115.33
Arctiidae	*Stigmatophora micans* (Bremer et Grey)	BHS110703.119	COI	BHS Mountain, Beijing	39.51	115.33
Arctiidae	*Stigmatophora micans* (Bremer et Grey)	BHS110703.044	COI	BHS Mountain, Beijing	39.51	115.33
Arctiidae	*Stigmatophora micans* (Bremer et Grey)	BHS110703.118	COI	BHS Mountain, Beijing	39.51	115.33
Arctiidae	*Stigmatophora micans* (Bremer et Grey)	BHS100703.244	COI	BHS Mountain, Beijing	39.51	115.33
Arctiidae	*Stigmatophora micans* (Bremer et Grey)	BHS100704.053	COI	BHS Mountain, Beijing	39.51	115.33
Arctiidae	*Stigmatophora micans* (Bremer et Grey)	BHS100704.065	COI	BHS Mountain, Beijing	39.51	115.33
Arctiidae	*Stigmatophora micans* (Bremer et Grey)	BHS100704.071	COI	BHS Mountain, Beijing	39.51	115.33
Arctiidae	*Stigmatophora micans* (Bremer et Grey)	BHS100704.237	COI	BHS Mountain, Beijing	39.51	115.33
Arctiidae	*Stigmatophora micans* (Bremer et Grey)	BHS100705.040	COI	BHS Mountain, Beijing	39.51	115.33
Arctiidae	*Stigmatophora micans* (Bremer et Grey)	BHS100705.112	COI	BHS Mountain, Beijing	39.51	115.33
Arctiidae	*Stigmatophora micans* (Bremer et Grey)	BHS100705.135	COI	BHS Mountain, Beijing	39.51	115.33
Arctiidae	*Stigmatophora rhodophila* (Walker)	BHS100703.175	COI	BHS Mountain, Beijing	39.51	115.33
Arctiidae	*Stigmatophora rhodophila* (Walker)	BHS100704.202	COI	BHS Mountain, Beijing	39.51	115.33
Arctiidae	*Stigmatophora rhodophila* (Walker)	BHS100705.100	COI	BHS Mountain, Beijing	39.51	115.33
Bombycidae	*Theophila mandarina* (Moore)	BHS110703.110	COI	BHS Mountain, Beijing	39.51	115.33
Bombycidae	*Theophila mandarina* (Moore)	BHS100704.203	COI	BHS Mountain, Beijing	39.51	115.33

Brahmaeidae	*Brahmaea christophi* (Staudinger)	BHS100703.308	COI	BHS Mountain, Beijing	39.51	115.33
Brahmaeidae	*Brahmaea christophi* (Staudinger)	BHS100703.311	COI	BHS Mountain, Beijing	39.51	115.33
Cossidae	*Zeuzera pyrina* (Staudinger et Rebel)	BHS100703.278	COI	BHS Mountain, Beijing	39.51	115.33
Cossidae	*Zeuzera pyrina* (Staudinger et Rebel)	BHS100703.307	COI	BHS Mountain, Beijing	39.51	115.33
Crambidae	*Anania verbascalis*	BHS100703.229	COI	BHS Mountain, Beijing	39.51	115.33
Crambidae	*Anania verbascalis*	BHS100703.117	COI	BHS Mountain, Beijing	39.51	115.33
Crambidae	*Anania verbascalis*	BHS100703.166	COI	BHS Mountain, Beijing	39.51	115.33
Crambidae	*Anania verbascalis*	BHS100704.138	COI	BHS Mountain, Beijing	39.51	115.33
Crambidae	*Anania verbascalis*	BHS100704.188	COI	BHS Mountain, Beijing	39.51	115.33
Crambidae	*Anania verbascalis*	BHS100704.245	COI	BHS Mountain, Beijing	39.51	115.33
Crambidae	*Circobotys heterogenalis* (Bremer, 1864)	BHS100703.183	COI	BHS Mountain, Beijing	39.51	115.33
Crambidae	*Circobotys heterogenalis* (Bremer, 1864)	BHS100703.223	COI	BHS Mountain, Beijing	39.51	115.33
Crambidae	*Circobotys heterogenalis* (Bremer, 1864)	BHS100703.243	COI	BHS Mountain, Beijing	39.51	115.33
Crambidae	*Circobotys heterogenalis* (Bremer, 1864)	BHS100703.248	COI	BHS Mountain, Beijing	39.51	115.33
Crambidae	*Circobotys heterogenalis* (Bremer, 1864)	BHS100704.162	COI	BHS Mountain, Beijing	39.51	115.33
Crambidae	*Circobotys heterogenalis* (Bremer, 1864)	BHS100705.108	COI	BHS Mountain, Beijing	39.51	115.33
Crambidae	*Cnaphalocrocis medinalis*	BHS100703.207	COI	BHS Mountain, Beijing	39.51	115.33
Crambidae	*Cnaphalocrocis medinalis*	BHS100705.143	COI	BHS Mountain, Beijing	39.51	115.33
Crambidae	*Diaphania quadrimaculalis*	BHS100703.111	COI	BHS Mountain, Beijing	39.51	115.33
Crambidae	*Diaphania quadrimaculalis*	BHS100704.179	COI	BHS Mountain, Beijing	39.51	115.33
Crambidae	*Diaphania quadrimaculalis*	BHS100704.190	COI	BHS Mountain, Beijing	39.51	115.33
Crambidae	*Diaphania quadrimaculalis*	BHS100705.031	COI	BHS Mountain, Beijing	39.51	115.33
Crambidae	*Eoophyla sinensis* (Hampson, 1897)	BHS100704.127	COI	BHS Mountain, Beijing	39.51	115.33
Crambidae	*Eoophyla sinensis* (Hampson, 1897)	BHS100705.049	COI	BHS Mountain, Beijing	39.51	115.33
Crambidae	*Euclasta stoetzneri* (Caradja, 1927)	BHS100705.116	COI	BHS Mountain, Beijing	39.51	115.33
Crambidae	*Euclasta stoetzneri* (Caradja, 1927)	BHS110703.139	COI	BHS Mountain, Beijing	39.51	115.33
Crambidae	*Herpetogramma luctuosalis* (Guenée, 1854)	BHS100703.126	COI	BHS Mountain, Beijing	39.51	115.33
Crambidae	*Herpetogramma luctuosalis* (Guenée, 1854)	BHS100703.150	COI	BHS Mountain, Beijing	39.51	115.33
Crambidae	*Herpetogramma luctuosalis* (Guenée, 1854)	BHS100703.156	COI	BHS Mountain, Beijing	39.51	115.33
Crambidae	*Herpetogramma luctuosalis* (Guenée, 1854)	BHS100704.113	COI	BHS Mountain, Beijing	39.51	115.33
Crambidae	*Lamprosema commixta* (Butler, 1879)	BHS100703.100	COI	BHS Mountain, Beijing	39.51	115.33
Crambidae	*Lamprosema commixta* (Butler, 1879)	BHS100703.152	COI	BHS Mountain, Beijing	39.51	115.33
Crambidae	*Lamprosema commixta* (Butler, 1879)	BHS100703.195	COI	BHS Mountain, Beijing	39.51	115.33
Crambidae	*Lamprosema commixta* (Butler, 1879)	BHS100703.200	COI	BHS Mountain, Beijing	39.51	115.33
Crambidae	*Lamprosema commixta* (Butler, 1879)	BHS100703.208	COI	BHS Mountain, Beijing	39.51	115.33
Crambidae	*Lamprosema commixta* (Butler, 1879)	BHS100703.210	COI	BHS Mountain, Beijing	39.51	115.33
Crambidae	*Lamprosema commixta* (Butler, 1879)	BHS100703.219	COI	BHS Mountain, Beijing	39.51	115.33
Crambidae	*Lamprosema commixta* (Butler, 1879)	BHS100704.171	COI	BHS Mountain, Beijing	39.51	115.33
Crambidae	*Lamprosema commixta* (Butler, 1879)	BHS100705.140	COI	BHS Mountain, Beijing	39.51	115.33
Crambidae	*Loxostege aeruginalis* (Hübner, 1796)	BHS100704.079	COI	BHS Mountain, Beijing	39.51	115.33
Crambidae	*Loxostege aeruginalis* (Hübner, 1796)	BHS100704.093	COI	BHS Mountain, Beijing	39.51	115.33
Crambidae	*Loxostege aeruginalis* (Hübner, 1796)	BHS100705.119	COI	BHS Mountain, Beijing	39.51	115.33
Crambidae	*Loxostege sticticalis* (Linnaeus, 1761)	BHS100704.082	COI	BHS Mountain, Beijing	39.51	115.33
Crambidae	*Loxostege sticticalis* (Linnaeus, 1761)	BHS100704.121	COI	BHS Mountain, Beijing	39.51	115.33
Crambidae	*Loxostege turbidalis* (Treitschke, 1829)	BHS100703.167	COI	BHS Mountain, Beijing	39.51	115.33
Crambidae	*Loxostege turbidalis* (Treitschke, 1829)	BHS100703.188	COI	BHS Mountain, Beijing	39.51	115.33
Crambidae	*Loxostege turbidalis* (Treitschke, 1829)	BHS100703.234	COI	BHS Mountain, Beijing	39.51	115.33
Crambidae	*Loxostege turbidalis* (Treitschke, 1829)	BHS100704.198	COI	BHS Mountain, Beijing	39.51	115.33
Crambidae	*Loxostege turbidalis* (Treitschke, 1829)	BHS100705.123	COI	BHS Mountain, Beijing	39.51	115.33
Crambidae	*Pleuroptya chlorophanta* (Butler, 1878)	BHS100703.084	COI	BHS Mountain, Beijing	39.51	115.33
Crambidae	*Pleuroptya chlorophanta* (Butler, 1878)	BHS100703.184	COI	BHS Mountain, Beijing	39.51	115.33
Crambidae	*Pleuroptya chlorophanta* (Butler, 1878)	BHS100703.214	COI	BHS Mountain, Beijing	39.51	115.33
Crambidae	*Pleuroptya chlorophanta* (Butler, 1878)	BHS100703.247	COI	BHS Mountain, Beijing	39.51	115.33
Crambidae	*Pleuroptya chlorophanta* (Butler, 1878)	BHS100703.267	COI	BHS Mountain, Beijing	39.51	115.33
Crambidae	*Pleuroptya chlorophanta* (Butler, 1878)	BHS100703.269	COI	BHS Mountain, Beijing	39.51	115.33
Crambidae	*Pleuroptya chlorophanta* (Butler, 1878)	BHS100704.086	COI	BHS Mountain, Beijing	39.51	115.33
Crambidae	*Pleuroptya chlorophanta* (Butler, 1878)	BHS100704.118	COI	BHS Mountain, Beijing	39.51	115.33
Crambidae	*Pleuroptya chlorophanta* (Butler, 1878)	BHS100704.143	COI	BHS Mountain, Beijing	39.51	115.33
Crambidae	*Pleuroptya chlorophanta* (Butler, 1878)	BHS100704.148	COI	BHS Mountain, Beijing	39.51	115.33
Crambidae	*Pleuroptya chlorophanta* (Butler, 1878)	BHS100704.149	COI	BHS Mountain, Beijing	39.51	115.33
Crambidae	*Pleuroptya chlorophanta* (Butler, 1878)	BHS100704.155	COI	BHS Mountain, Beijing	39.51	115.33
Crambidae	*Pleuroptya chlorophanta* (Butler, 1878)	BHS100704.193	COI	BHS Mountain, Beijing	39.51	115.33
Crambidae	*Pleuroptya chlorophanta* (Butler, 1878)	BHS100704.197	COI	BHS Mountain, Beijing	39.51	115.33
Crambidae	*Pleuroptya chlorophanta* (Butler, 1878)	BHS100704.218	COI	BHS Mountain, Beijing	39.51	115.33
Crambidae	*Pleuroptya chlorophanta* (Butler, 1878)	BHS100705.082	COI	BHS Mountain, Beijing	39.51	115.33
Crambidae	*Pleuroptya chlorophanta* (Butler, 1878)	BHS100705.088	COI	BHS Mountain, Beijing	39.51	115.33

Crambidae	*Pleuroptya chlorophanta* (Butler, 1878)	BHS100705.098	COI	BHS Mountain, Beijing	39.51	115.33
Crambidae	*Pleuroptya chlorophanta* (Butler, 1878)	BHS100705.101	COI	BHS Mountain, Beijing	39.51	115.33
Crambidae	*Pleuroptya chlorophanta* (Butler, 1878)	BHS100705.107	COI	BHS Mountain, Beijing	39.51	115.33
Crambidae	*Pleuroptya chlorophanta* (Butler, 1878)	BHS100705.133	COI	BHS Mountain, Beijing	39.51	115.33
Crambidae	*Pleuroptya chlorophanta* (Butler, 1878)	BHS110703.047	COI	BHS Mountain, Beijing	39.51	115.33
Crambidae	*Sitochroa verticalis* (Linnaeus, 1758)	BHS100703.085	COI	BHS Mountain, Beijing	39.51	115.33
Crambidae	*Sitochroa verticalis* (Linnaeus, 1758)	BHS100703.087	COI	BHS Mountain, Beijing	39.51	115.33
Crambidae	*Sitochroa verticalis* (Linnaeus, 1758)	BHS100703.089	COI	BHS Mountain, Beijing	39.51	115.33
Crambidae	*Sitochroa verticalis* (Linnaeus, 1758)	BHS100703.103	COI	BHS Mountain, Beijing	39.51	115.33
Crambidae	*Sitochroa verticalis* (Linnaeus, 1758)	BHS100703.139	COI	BHS Mountain, Beijing	39.51	115.33
Crambidae	*Sitochroa verticalis* (Linnaeus, 1758)	BHS100703.174	COI	BHS Mountain, Beijing	39.51	115.33
Crambidae	*Sitochroa verticalis* (Linnaeus, 1758)	BHS100703.176	COI	BHS Mountain, Beijing	39.51	115.33
Crambidae	*Sitochroa verticalis* (Linnaeus, 1758)	BHS100703.189	COI	BHS Mountain, Beijing	39.51	115.33
Crambidae	*Sitochroa verticalis* (Linnaeus, 1758)	BHS100703.258	COI	BHS Mountain, Beijing	39.51	115.33
Crambidae	*Sitochroa verticalis* (Linnaeus, 1758)	BHS100703.259	COI	BHS Mountain, Beijing	39.51	115.33
Crambidae	*Sitochroa verticalis* (Linnaeus, 1758)	BHS100703.263	COI	BHS Mountain, Beijing	39.51	115.33
Crambidae	*Sitochroa verticalis* (Linnaeus, 1758)	BHS100704.003	COI	BHS Mountain, Beijing	39.51	115.33
Crambidae	*Sitochroa verticalis* (Linnaeus, 1758)	BHS100704.010	COI	BHS Mountain, Beijing	39.51	115.33
Crambidae	*Sitochroa verticalis* (Linnaeus, 1758)	BHS100704.111	COI	BHS Mountain, Beijing	39.51	115.33
Crambidae	*Sitochroa verticalis* (Linnaeus, 1758)	BHS100704.211	COI	BHS Mountain, Beijing	39.51	115.33
Crambidae	*Sitochroa verticalis* (Linnaeus, 1758)	BHS100705.084	COI	BHS Mountain, Beijing	39.51	115.33
Crambidae	*Sitochroa verticalis* (Linnaeus, 1758)	BHS100705.097	COI	BHS Mountain, Beijing	39.51	115.33
Crambidae	*Sitochroa verticalis* (Linnaeus, 1758)	BHS100705.111	COI	BHS Mountain, Beijing	39.51	115.33
Crambidae	*Sitochroa verticalis* (Linnaeus, 1758)	BHS100705.118	COI	BHS Mountain, Beijing	39.51	115.33
Crambidae	*Sitochroa verticalis* (Linnaeus, 1758)	BHS100705.139	COI	BHS Mountain, Beijing	39.51	115.33
Crambidae	*Stenia charonialis* (Walker, 1859)	BHS100704.185	COI	BHS Mountain, Beijing	39.51	115.33
Crambidae	*Stenia charonialis* (Walker, 1859)	BHS100704.213	COI	BHS Mountain, Beijing	39.51	115.33
Crambidae	*Stenia charonialis* (Walker, 1859)	BHS100705.121	COI	BHS Mountain, Beijing	39.51	115.33
Lasiocampida	*Dendrolimus tabulaeformis* (Tsai et Liu)	BHS100703.285	COI	BHS Mountain, Beijing	39.51	115.33
Lasiocampida	*Dendrolimus tabulaeformis* (Tsai et Liu)	BHS100703.297	COI	BHS Mountain, Beijing	39.51	115.33
Limacodidae	*Narosoideus flavidorsalis* (Staudinger, 1887)	BHS110703.004	COI	BHS Mountain, Beijing	39.51	115.33
Limacodidae	*Narosoideus flavidorsalis* (Staudinger, 1887)	BHS110703.008	COI	BHS Mountain, Beijing	39.51	115.33
Limacodidae	*Narosoideus flavidorsalis* (Staudinger, 1887)	BHS100703.097	COI	BHS Mountain, Beijing	39.51	115.33
Limacodidae	*Narosoideus flavidorsalis* (Staudinger, 1887)	BHS100703.101	COI	BHS Mountain, Beijing	39.51	115.33
Limacodidae	*Narosoideus flavidorsalis* (Staudinger, 1887)	BHS100703.108	COI	BHS Mountain, Beijing	39.51	115.33
Limacodidae	*Narosoideus flavidorsalis* (Staudinger, 1887)	BHS100703.123	COI	BHS Mountain, Beijing	39.51	115.33
Limacodidae	*Narosoideus flavidorsalis* (Staudinger, 1887)	BHS100703.130	COI	BHS Mountain, Beijing	39.51	115.33
Limacodidae	*Narosoideus flavidorsalis* (Staudinger, 1887)	BHS100703.132	COI	BHS Mountain, Beijing	39.51	115.33
Limacodidae	*Narosoideus flavidorsalis* (Staudinger, 1887)	BHS100703.288	COI	BHS Mountain, Beijing	39.51	115.33
Limacodidae	*Narosoideus flavidorsalis* (Staudinger, 1887)	BHS100704.008	COI	BHS Mountain, Beijing	39.51	115.33
Limacodidae	*Narosoideus flavidorsalis* (Staudinger, 1887)	BHS100704.037	COI	BHS Mountain, Beijing	39.51	115.33
Lymantriidae	*Narosoideus flavidorsalis* (Staudinger, 1887)	BHS110703.021	COI	BHS Mountain, Beijing	39.51	115.33
Lymantriidae	*Narosoideus flavidorsalis* (Staudinger, 1887)	BHS110703.071	COI	BHS Mountain, Beijing	39.51	115.33
Lymantriidae	*Narosoideus flavidorsalis* (Staudinger, 1887)	BHS110703.080	COI	BHS Mountain, Beijing	39.51	115.33
Limacodidae	*Narosoideus flavidorsalis* (Staudinger, 1887)	BHS100704.206	COI	BHS Mountain, Beijing	39.51	115.33
Limacodidae	*Narosoideus flavidorsalis* (Staudinger, 1887)	BHS100705.026	COI	BHS Mountain, Beijing	39.51	115.33
Limacodidae	*Narosoideus flavidorsalis* (Staudinger, 1887)	BHS100705.054	COI	BHS Mountain, Beijing	39.51	115.33
Limacodidae	*Parasa consocia* (Walker, 1965)	BHS100703.294	COI	BHS Mountain, Beijing	39.51	115.33
Limacodidae	*Parasa consocia* (Walker, 1965)	BHS100704.059	COI	BHS Mountain, Beijing	39.51	115.33
Limacodidae	*Parasa consocia* (Walker, 1965)	BHS100704.081	COI	BHS Mountain, Beijing	39.51	115.33
Limacodidae	*Parasa consocia* (Walker, 1965)	BHS100704.110	COI	BHS Mountain, Beijing	39.51	115.33
Limacodidae	*Parasa consocia* (Walker, 1965)	BHS100704.130	COI	BHS Mountain, Beijing	39.51	115.33
Limacodidae	*Parasa consocia* (Walker, 1965)	BHS100704.144	COI	BHS Mountain, Beijing	39.51	115.33
Limacodidae	*Parasa consocia* (Walker, 1965)	BHS100704.156	COI	BHS Mountain, Beijing	39.51	115.33
Limacodidae	*Parasa consocia* (Walker, 1965)	BHS100704.207	COI	BHS Mountain, Beijing	39.51	115.33
Limacodidae	*Parasa consocia* (Walker, 1965)	BHS100704.228	COI	BHS Mountain, Beijing	39.51	115.33
Limacodidae	*Parasa consocia* (Walker, 1965)	BHS100705.038	COI	BHS Mountain, Beijing	39.51	115.33
Limacodidae	*Parasa consocia* (Walker, 1965)	BHS100705.081	COI	BHS Mountain, Beijing	39.51	115.33
Limacodidae	*Parasa consocia* (Walker, 1965)	BHS100705.096	COI	BHS Mountain, Beijing	39.51	115.33
Limacodidae	*Thosea sinensis* (Walker, 1855)	BHS100703.046	COI	BHS Mountain, Beijing	39.51	115.33
Limacodidae	*Thosea sinensis* (Walker, 1855)	BHS100703.135	COI	BHS Mountain, Beijing	39.51	115.33
Limacodidae	*Thosea sinensis* (Walker, 1855)	BHS100703.225	COI	BHS Mountain, Beijing	39.51	115.33
Limacodidae	*Thosea sinensis* (Walker, 1855)	BHS100704.014	COI	BHS Mountain, Beijing	39.51	115.33
Limacodidae	*Thosea sinensis* (Walker, 1855)	BHS100704.153	COI	BHS Mountain, Beijing	39.51	115.33
Limacodidae	*Thosea sinensis* (Walker, 1855)	BHS100705.070	COI	BHS Mountain, Beijing	39.51	115.33
Lymantriidae	*Kuromondokuga niphonis*	BHS100704.001	COI	BHS Mountain, Beijing	39.51	115.33

Lymantriidae	*Kuromondokuga niphonis*	BHS100705.080	COI	BHS Mountain, Beijing	39.51	115.33
Lymantriidae	*Sphrageidus similis*	BHS100703.074	COI	BHS Mountain, Beijing	39.51	115.33
Lymantriidae	*Sphrageidus similis*	BHS100703.121	COI	BHS Mountain, Beijing	39.51	115.33
Lymantriidae	*Sphrageidus similis*	BHS100703.271	COI	BHS Mountain, Beijing	39.51	115.33
Lymantriidae	*Sphrageidus similis*	BHS100704.186	COI	BHS Mountain, Beijing	39.51	115.33
Lymantriidae	*Sphrageidus similis*	BHS100705.105	COI	BHS Mountain, Beijing	39.51	115.33
Lymantriidae	*Stilpnotia candida* (Staudinger)	BHS100703.082	COI	BHS Mountain, Beijing	39.51	115.33
Lymantriidae	*Stilpnotia candida* (Staudinger)	BHS100703.120	COI	BHS Mountain, Beijing	39.51	115.33
Lymantriidae	*Teia parallela*	BHS100703.044	COI	BHS Mountain, Beijing	39.51	115.33
Lymantriidae	*Teia parallela*	BHS100703.058	COI	BHS Mountain, Beijing	39.51	115.33
Lymantriidae	*Teia parallela*	BHS100703.059	COI	BHS Mountain, Beijing	39.51	115.33
Lymantriidae	*Teia parallela*	BHS100703.112	COI	BHS Mountain, Beijing	39.51	115.33
Lymantriidae	*Teia parallela*	BHS100704.035	COI	BHS Mountain, Beijing	39.51	115.33
Lymantriidae	*Teia parallela*	BHS100704.100	COI	BHS Mountain, Beijing	39.51	115.33
Lymantriidae	*Teia parallela*	BHS100704.134	COI	BHS Mountain, Beijing	39.51	115.33
Lymantriidae	*Teia parallela*	BHS100704.168	COI	BHS Mountain, Beijing	39.51	115.33
Lymantriidae	*Teia parallela*	BHS100705.034	COI	BHS Mountain, Beijing	39.51	115.33
Lymantriidae	*Teia parallela*	BHS100705.050	COI	BHS Mountain, Beijing	39.51	115.33
Lymantriidae	*Teia parallela*	BHS100705.051	COI	BHS Mountain, Beijing	39.51	115.33
Lymantriidae	*Teia parallela*	BHS100705.056	COI	BHS Mountain, Beijing	39.51	115.33
Lymantriidae	*Teia parallela*	BHS100705.086	COI	BHS Mountain, Beijing	39.51	115.33
Lymantriidae	*Teia parallela*	BHS100705.087	COI	BHS Mountain, Beijing	39.51	115.33
Lymantriidae	*Teia parallela*	BHS100705.093	COI	BHS Mountain, Beijing	39.51	115.33
Lymantriidae	*Teia parallela*	BHS110703.009	COI	BHS Mountain, Beijing	39.51	115.33
Lymantriidae	*Teia parallela*	BHS110703.028	COI	BHS Mountain, Beijing	39.51	115.33
Lymantriidae	*Teia parallela*	BHS110703.049	COI	BHS Mountain, Beijing	39.51	115.33
Lymantriidae	*Teia parallela*	BHS110703.143	COI	BHS Mountain, Beijing	39.51	115.33
Noctuidae	*Athetis lineosa* (Moore, 1881)	BHS100704.017	COI	BHS Mountain, Beijing	39.51	115.33
Noctuidae	*Athetis lineosa* (Moore, 1881)	BHS100704.073	COI	BHS Mountain, Beijing	39.51	115.33
Noctuidae	*Callopistria juventina* (Stoll, 1782)	BHS100703.045	COI	BHS Mountain, Beijing	39.51	115.33
Noctuidae	*Callopistria juventina* (Stoll, 1782)	BHS100703.099	COI	BHS Mountain, Beijing	39.51	115.33
Noctuidae	*Callopistria juventina* (Stoll, 1782)	BHS100703.171	COI	BHS Mountain, Beijing	39.51	115.33
Noctuidae	*Callopistria juventina* (Stoll, 1782)	BHS100703.201	COI	BHS Mountain, Beijing	39.51	115.33
Noctuidae	*Callopistria juventina* (Stoll, 1782)	BHS100705.132	COI	BHS Mountain, Beijing	39.51	115.33
Noctuidae	*Catocala columbina* (Leech, 1900)	BHS100703.295	COI	BHS Mountain, Beijing	39.51	115.33
Noctuidae	*Catocala columbina* (Leech, 1900)	BHS100703.306	COI	BHS Mountain, Beijing	39.51	115.33
Noctuidae	*Chrysorithrum amatum* (Bremer & Grey, 1853)	BHS100704.033	COI	BHS Mountain, Beijing	39.51	115.33
Noctuidae	*Chrysorithrum amatum* (Bremer & Grey, 1853)	BHS100704.078	COI	BHS Mountain, Beijing	39.51	115.33
Noctuidae	*Flexivaleria mienshani* (Draudt, 1950)	BHS100703.093	COI	BHS Mountain, Beijing	39.51	115.33
Noctuidae	*Flexivaleria mienshani* (Draudt, 1950)	BHS100704.150	COI	BHS Mountain, Beijing	39.51	115.33
Noctuidae	*Flexivaleria mienshani* (Draudt, 1950)	BHS100705.033	COI	BHS Mountain, Beijing	39.51	115.33
Noctuidae	*Hadjina chinensis* (Wallengren, 1860)	BHS100703.102	COI	BHS Mountain, Beijing	39.51	115.33
Noctuidae	*Hadjina chinensis* (Wallengren, 1860)	BHS100703.127	COI	BHS Mountain, Beijing	39.51	115.33
Noctuidae	*Hadjina chinensis* (Wallengren, 1860)	BHS100703.131	COI	BHS Mountain, Beijing	39.51	115.33
Noctuidae	*Hadjina chinensis* (Wallengren, 1860)	BHS100703.270	COI	BHS Mountain, Beijing	39.51	115.33
Noctuidae	*Hadjina chinensis* (Wallengren, 1860)	BHS100704.181	COI	BHS Mountain, Beijing	39.51	115.33
Noctuidae	*Hadjina chinensis* (Wallengren, 1860)	BHS100704.209	COI	BHS Mountain, Beijing	39.51	115.33
Noctuidae	*Hadjina chinensis* (Wallengren, 1860)	BHS100704.222	COI	BHS Mountain, Beijing	39.51	115.33
Noctuidae	*Hadjina chinensis* (Wallengren, 1860)	BHS100704.233	COI	BHS Mountain, Beijing	39.51	115.33
Noctuidae	*Hadjina chinensis* (Wallengren, 1860)	BHS100705.117	COI	BHS Mountain, Beijing	39.51	115.33
Noctuidae	*Hadjina chinensis* (Wallengren, 1860)	BHS100705.136	COI	BHS Mountain, Beijing	39.51	115.33
Noctuidae	*Hypena tristalis* (Lederer,1853)	BHS100704.002	COI	BHS Mountain, Beijing	39.51	115.33
Noctuidae	*Hypena tristalis* (Lederer,1853)	BHS100705.023	COI	BHS Mountain, Beijing	39.51	115.33
Noctuidae	*Hypena tristalis* (Lederer,1853)	BHS100705.066	COI	BHS Mountain, Beijing	39.51	115.33
Noctuidae	*Hypena tristalis* (Lederer,1853)	BHS100705.124	COI	BHS Mountain, Beijing	39.51	115.33
Noctuidae	*Hypena tristalis* (Lederer,1853)	BHS100703.096	COI	BHS Mountain, Beijing	39.51	115.33
Noctuidae	*Hypena(Bomolocha) stygiana* (Butler, 1878)	BHS100703.072	COI	BHS Mountain, Beijing	39.51	115.33
Noctuidae	*Hypena(Bomolocha) stygiana* (Butler, 1878)	BHS100703.246	COI	BHS Mountain, Beijing	39.51	115.33
Noctuidae	*Hypocala subsatura* (Guenee, 1852)	BHS100703.119	COI	BHS Mountain, Beijing	39.51	115.33
Noctuidae	*Hypocala subsatura* (Guenee, 1852)	BHS100705.035	COI	BHS Mountain, Beijing	39.51	115.33
Noctuidae	*Lacanobia aliena* (Hübner, 1807)	BHS100703.024	COI	BHS Mountain, Beijing	39.51	115.33
Noctuidae	*Lacanobia aliena* (Hübner, 1807)	BHS100704.132	COI	BHS Mountain, Beijing	39.51	115.33
Noctuidae	*Lacanobia aliena* (Hübner, 1807)	BHS100704.151	COI	BHS Mountain, Beijing	39.51	115.33
Noctuidae	*Lacanobia aliena* (Hübner, 1807)	BHS100705.085	COI	BHS Mountain, Beijing	39.51	115.33
Noctuidae	*Lacanobia aliena* (Hübner, 1807)	BHS110703.010	COI	BHS Mountain, Beijing	39.51	115.33
Noctuidae	*Lacanobia aliena* (Hübner, 1807)	BHS110703.112	COI	BHS Mountain, Beijing	39.51	115.33

Noctuidae	*Niphonyx segregata* (Butler, 1878)	BHS100703.161	COI	BHS Mountain, Beijing	39.51	115.33
Noctuidae	*Niphonyx segregata* (Butler, 1878)	BHS100703.218	COI	BHS Mountain, Beijing	39.51	115.33
Noctuidae	*Niphonyx segregata* (Butler, 1878)	BHS100704.223	COI	BHS Mountain, Beijing	39.51	115.33
Noctuidae	*Pangrapta disruptalis* (Walker, 1865)	BHS100704.216	COI	BHS Mountain, Beijing	39.51	115.33
Noctuidae	*Pangrapta disruptalis* (Walker, 1865)	BHS100705.061	COI	BHS Mountain, Beijing	39.51	115.33
Noctuidae	*Paracolax derivalis*	BHS110703.029	COI	BHS Mountain, Beijing	39.51	115.33
Noctuidae	*Paracolax derivalis*	BHS110703.059	COI	BHS Mountain, Beijing	39.51	115.33
Noctuidae	*Phyllophila obliterata* (Rambur, 1833)	BHS100703.237	COI	BHS Mountain, Beijing	39.51	115.33
Noctuidae	*Phyllophila obliterata* (Rambur, 1833)	BHS100704.096	COI	BHS Mountain, Beijing	39.51	115.33
Noctuidae	*Phyllophila obliterata* (Rambur, 1833)	BHS110703.138	COI	BHS Mountain, Beijing	39.51	115.33
Noctuidae	*Xanthomantis cornelia* (Staudinger, 1888)	BHS100704.016	COI	BHS Mountain, Beijing	39.51	115.33
Noctuidae	*Xanthomantis cornelia* (Staudinger, 1888)	BHS100704.074	COI	BHS Mountain, Beijing	39.51	115.33
Noctuidae	*Xestia(Megasema) ditrapezium* (Denis & Schiffermuller, 1775)	BHS100704.075	COI	BHS Mountain, Beijing	39.51	115.33
Noctuidae	*Xestia(Megasema) ditrapezium* (Denis & Schiffermuller, 1775)	BHS100704.085	COI	BHS Mountain, Beijing	39.51	115.33
Noctuidae	*Zanclognatha lunalis* (Scopoli, 1763)	BHS100703.060	COI	BHS Mountain, Beijing	39.51	115.33
Noctuidae	*Zanclognatha lunalis* (Scopoli, 1763)	BHS100704.120	COI	BHS Mountain, Beijing	39.51	115.33
Notodontidae	*Clostera albosigma curtuloides* (Erschoff,1870)	BHS100704.131	COI	BHS Mountain, Beijing	39.51	115.33
Notodontidae	*Clostera albosigma curtuloides* (Erschoff,1870)	BHS100704.201	COI	BHS Mountain, Beijing	39.51	115.33
Notodontidae	*Gluphisia crenata meridionalis* (Kiriakoff)	BHS100703.128	COI	BHS Mountain, Beijing	39.51	115.33
Notodontidae	*Gluphisia crenata meridionalis* (Kiriakoff)	BHS100704.039	COI	BHS Mountain, Beijing	39.51	115.33
Notodontidae	*Gluphisia crenata meridionalis* (Kiriakoff)	BHS100704.164	COI	BHS Mountain, Beijing	39.51	115.33
Notodontidae	*Gluphisia crenata meridionalis* (Kiriakoff)	BHS100704.187	COI	BHS Mountain, Beijing	39.51	115.33
Notodontidae	*Lophocosma nigrilinea* (Leech)	BHS100703.030	COI	BHS Mountain, Beijing	39.51	115.33
Notodontidae	*Lophocosma nigrilinea* (Leech)	BHS100704.060	COI	BHS Mountain, Beijing	39.51	115.33
Notodontidae	*Micromelalopha sieversi* (Staudinger, 1892)	BHS100703.157	COI	BHS Mountain, Beijing	39.51	115.33
Notodontidae	*Micromelalopha sieversi* (Staudinger, 1892)	BHS100703.181	COI	BHS Mountain, Beijing	39.51	115.33
Notodontidae	*Micromelalopha sieversi* (Staudinger, 1892)	BHS100703.257	COI	BHS Mountain, Beijing	39.51	115.33
Notodontidae	*Micromelalopha sieversi* (Staudinger, 1892)	BHS100704.046	COI	BHS Mountain, Beijing	39.51	115.33
Notodontidae	*Micromelalopha sieversi* (Staudinger, 1892)	BHS100704.050	COI	BHS Mountain, Beijing	39.51	115.33
Notodontidae	*Micromelalopha sieversi* (Staudinger, 1892)	BHS100704.052	COI	BHS Mountain, Beijing	39.51	115.33
Notodontidae	*Micromelalopha sieversi* (Staudinger, 1892)	BHS100704.159	COI	BHS Mountain, Beijing	39.51	115.33
Notodontidae	*Micromelalopha sieversi* (Staudinger, 1892)	BHS100704.160	COI	BHS Mountain, Beijing	39.51	115.33
Notodontidae	*Micromelalopha sieversi* (Staudinger, 1892)	BHS100704.182	COI	BHS Mountain, Beijing	39.51	115.33
Notodontidae	*Micromelalopha sieversi* (Staudinger, 1892)	BHS100704.195	COI	BHS Mountain, Beijing	39.51	115.33
Notodontidae	*Micromelalopha sieversi* (Staudinger, 1892)	BHS100705.091	COI	BHS Mountain, Beijing	39.51	115.33
Notodontidae	*Nerice hoenei* (Kiriakoff)	BHS100703.277	COI	BHS Mountain, Beijing	39.51	115.33
Notodontidae	*Nerice hoenei* (Kiriakoff)	BHS100705.008	COI	BHS Mountain, Beijing	39.51	115.33
Notodontidae	*Nerice hoenei* (Kiriakoff)	BHS100705.017	COI	BHS Mountain, Beijing	39.51	115.33
Notodontidae	*Stauropus basalis* (Moore)	BHS100703.284	COI	BHS Mountain, Beijing	39.51	115.33
Notodontidae	*Stauropus basalis* (Moore)	BHS100704.057	COI	BHS Mountain, Beijing	39.51	115.33
Notodontidae	*Stauropus basalis* (Moore)	BHS100704.126	COI	BHS Mountain, Beijing	39.51	115.33
Notodontidae	*Stauropus basalis* (Moore)	BHS100704.167	COI	BHS Mountain, Beijing	39.51	115.33
Pyralidae	*Epilepia dentata*	BHS100704.098	COI	BHS Mountain, Beijing	39.51	115.33
Pyralidae	*Epilepia dentata*	BHS100704.189	COI	BHS Mountain, Beijing	39.51	115.33
Pyralidae	*Glyptoteles leucacrinella*	BHS100703.151	COI	BHS Mountain, Beijing	39.51	115.33
Pyralidae	*Glyptoteles leucacrinella*	BHS100703.164	COI	BHS Mountain, Beijing	39.51	115.33
Pyralidae	*Glyptoteles leucacrinella*	BHS100703.178	COI	BHS Mountain, Beijing	39.51	115.33
Pyralidae	*Nephopterix shantungella* (Roseler, 1969)	BHS100703.148	COI	BHS Mountain, Beijing	39.51	115.33
Pyralidae	*Nephopterix shantungella* (Roseler, 1969)	BHS100703.179	COI	BHS Mountain, Beijing	39.51	115.33
Pyralidae	*Nephopterix shantungella* (Roseler, 1969)	BHS100704.192	COI	BHS Mountain, Beijing	39.51	115.33
Pyralidae	*Oncocera semirubella* (Scopoli, 1763)	BHS100703.198	COI	BHS Mountain, Beijing	39.51	115.33
Pyralidae	*Oncocera semirubella* (Scopoli, 1763)	BHS100705.125	COI	BHS Mountain, Beijing	39.51	115.33
Pyralidae	*Oncocera semirubella* (Scopoli, 1763)	BHS100705.130	COI	BHS Mountain, Beijing	39.51	115.33
Pyralidae	*Pyralis regalis* (Schiffermüller et Denis, 1775)	BHS100703.206	COI	BHS Mountain, Beijing	39.51	115.33
Pyralidae	*Pyralis regalis* (Schiffermüller et Denis, 1775)	BHS100703.281	COI	BHS Mountain, Beijing	39.51	115.33
Pyralidae	*Pyralis regalis* (Schiffermüller et Denis, 1775)	BHS100704.080	COI	BHS Mountain, Beijing	39.51	115.33
Pyralidae	*Teliphasa elegans* (Butler, 1881)	BHS100704.249	COI	BHS Mountain, Beijing	39.51	115.33
Pyralidae	*Teliphasa elegans* (Butler, 1881)	BHS100705.030	COI	BHS Mountain, Beijing	39.51	115.33
Pyralidae	*Teliphasa elegans* (Butler, 1881)	BHS100705.042	COI	BHS Mountain, Beijing	39.51	115.33
Pyralidae	*Termioptycha nigrescens* (Warren, 1891)	BHS100703.241	COI	BHS Mountain, Beijing	39.51	115.33
Pyralidae	*Termioptycha nigrescens* (Warren, 1891)	BHS100704.146	COI	BHS Mountain, Beijing	39.51	115.33
Pyralidae	*Termioptycha nigrescens* (Warren, 1891)	BHS100704.177	COI	BHS Mountain, Beijing	39.51	115.33
Pyralidae	*Termioptycha nigrescens* (Warren, 1891)	BHS100705.114	COI	BHS Mountain, Beijing	39.51	115.33
Sphingidae	*Ambulyx ochracea* (Bulter)	BHS100703.001	COI	BHS Mountain, Beijing	39.51	115.33
Sphingidae	*Ambulyx ochracea* (Bulter)	BHS100703.040	COI	BHS Mountain, Beijing	39.51	115.33

Sphingidae	*Ambulyx ochracea* (Bulter)	BHS100704.020	COI	BHS Mountain, Beijing	39.51	115.33
Sphingidae	*Ambulyx ochracea* (Bulter)	BHS100704.028	COI	BHS Mountain, Beijing	39.51	115.33
Sphingidae	*Ambulyx ochracea* (Bulter)	BHS100704.032	COI	BHS Mountain, Beijing	39.51	115.33
Sphingidae	*Ambulyx ochracea* (Bulter)	BHS100705.021	COI	BHS Mountain, Beijing	39.51	115.33
Sphingidae	*Amorpha amurensis* (Staudinger)	BHS100703.296	COI	BHS Mountain, Beijing	39.51	115.33
Sphingidae	*Amorpha amurensis* (Staudinger)	BHS100703.299	COI	BHS Mountain, Beijing	39.51	115.33
Sphingidae	*Ampelophaga rubiginosa* (Bremer et Grey)	BHS100704.022	COI	BHS Mountain, Beijing	39.51	115.33
Sphingidae	*Ampelophaga rubiginosa* (Bremer et Grey)	BHS100704.023	COI	BHS Mountain, Beijing	39.51	115.33
Sphingidae	*Ampelophaga rubiginosa* (Bremer et Grey)	BHS100704.025	COI	BHS Mountain, Beijing	39.51	115.33
Sphingidae	*Ampelophaga rubiginosa* (Bremer et Grey)	BHS100704.026	COI	BHS Mountain, Beijing	39.51	115.33
Sphingidae	*Ampelophaga rubiginosa* (Bremer et Grey)	BHS100704.029	COI	BHS Mountain, Beijing	39.51	115.33
Sphingidae	*Ampelophaga rubiginosa* (Bremer et Grey)	BHS100705.002	COI	BHS Mountain, Beijing	39.51	115.33
Sphingidae	*Ampelophaga rubiginosa* (Bremer et Grey)	BHS100705.006	COI	BHS Mountain, Beijing	39.51	115.33
Sphingidae	*Ampelophaga rubiginosa* (Bremer et Grey)	BHS100705.011	COI	BHS Mountain, Beijing	39.51	115.33
Sphingidae	*Ampelophaga rubiginosa* (Bremer et Grey)	BHS100705.016	COI	BHS Mountain, Beijing	39.51	115.33
Sphingidae	*Ampelophaga rubiginosa* (Bremer et Grey)	BHS100705.019	COI	BHS Mountain, Beijing	39.51	115.33
Sphingidae	*Callambulyx tatarinovi* (Bremer et Grey)	BHS100703.002	COI	BHS Mountain, Beijing	39.51	115.33
Sphingidae	*Callambulyx tatarinovi* (Bremer et Grey)	BHS100703.012	COI	BHS Mountain, Beijing	39.51	115.33
Sphingidae	*Callambulyx tatarinovi* (Bremer et Grey)	BHS100703.015	COI	BHS Mountain, Beijing	39.51	115.33
Sphingidae	*Callambulyx tatarinovi* (Bremer et Grey)	BHS100703.017	COI	BHS Mountain, Beijing	39.51	115.33
Sphingidae	*Callambulyx tatarinovi* (Bremer et Grey)	BHS100703.039	COI	BHS Mountain, Beijing	39.51	115.33
Sphingidae	*Callambulyx tatarinovi* (Bremer et Grey)	BHS100703.041	COI	BHS Mountain, Beijing	39.51	115.33
Sphingidae	*Callambulyx tatarinovi* (Bremer et Grey)	BHS100703.264	COI	BHS Mountain, Beijing	39.51	115.33
Sphingidae	*Callambulyx tatarinovi* (Bremer et Grey)	BHS100703.301	COI	BHS Mountain, Beijing	39.51	115.33
Sphingidae	*Callambulyx tatarinovi* (Bremer et Grey)	BHS100704.021	COI	BHS Mountain, Beijing	39.51	115.33
Sphingidae	*Callambulyx tatarinovi* (Bremer et Grey)	BHS100705.004	COI	BHS Mountain, Beijing	39.51	115.33
Sphingidae	*Callambulyx tatarinovi* (Bremer et Grey)	BHS100705.009	COI	BHS Mountain, Beijing	39.51	115.33
Sphingidae	*Callambulyx tatarinovi* (Bremer et Grey)	BHS100705.013	COI	BHS Mountain, Beijing	39.51	115.33
Sphingidae	*Callambulyx tatarinovi* (Bremer et Grey)	BHS110703.079	COI	BHS Mountain, Beijing	39.51	115.33
Sphingidae	*Callambulyx tatarinovi* (Bremer et Grey)	BHS110703.151	COI	BHS Mountain, Beijing	39.51	115.33
Sphingidae	*Callambulyx tatarinovi* (Bremer et Grey)	BHS110703.154	COI	BHS Mountain, Beijing	39.51	115.33
Sphingidae	*Callambulyx tatarinovi* (Bremer et Grey)	BHS110703.155	COI	BHS Mountain, Beijing	39.51	115.33
Sphingidae	*Callambulyx tatarinovi* (Bremer et Grey)	BHS110703.156	COI	BHS Mountain, Beijing	39.51	115.33
Sphingidae	*Callambulyx tatarinovi* (Bremer et Grey)	BHS110703.160	COI	BHS Mountain, Beijing	39.51	115.33
Sphingidae	*Callambulyx tatarinovi* (Bremer et Grey)	BHS110703.165	COI	BHS Mountain, Beijing	39.51	115.33
Sphingidae	*Callambulyx tatarinovi* (Bremer et Grey)	BHS110703.168	COI	BHS Mountain, Beijing	39.51	115.33
Sphingidae	*Kentrochrysalis sieversi* (Alphéraky)	BHS100703.003	COI	BHS Mountain, Beijing	39.51	115.33
Sphingidae	*Kentrochrysalis sieversi* (Alphéraky)	BHS100703.038	COI	BHS Mountain, Beijing	39.51	115.33
Sphingidae	*Kentrochrysalis sieversi* (Alphéraky)	BHS100704.019	COI	BHS Mountain, Beijing	39.51	115.33
Sphingidae	*Kentrochrysalis sieversi* (Alphéraky)	BHS100704.027	COI	BHS Mountain, Beijing	39.51	115.33
Sphingidae	*Kentrochrysalis sieversi* (Alphéraky)	BHS100704.031	COI	BHS Mountain, Beijing	39.51	115.33
Sphingidae	*Kentrochrysalis sieversi* (Alphéraky)	BHS100705.007	COI	BHS Mountain, Beijing	39.51	115.33
Sphingidae	*Kentrochrysalis sieversi* (Alphéraky)	BHS100705.014	COI	BHS Mountain, Beijing	39.51	115.33
Sphingidae	*Marumba maacki* (Bremer)	BHS100703.042	COI	BHS Mountain, Beijing	39.51	115.33
Sphingidae	*Marumba maacki* (Bremer)	BHS100705.012	COI	BHS Mountain, Beijing	39.51	115.33
Sphingidae	*Marumba gaschkewitschi* (Bremer et Grey)	BHS100703.011	COI	BHS Mountain, Beijing	39.51	115.33
Sphingidae	*Marumba gaschkewitschi* (Bremer et Grey)	BHS100705.015	COI	BHS Mountain, Beijing	39.51	115.33
Sphingidae	*Marumba gaschkewitschi* (Bremer et Grey)	BHS110703.153	COI	BHS Mountain, Beijing	39.51	115.33
Sphingidae	*Marumba gaschkewitschi* (Bremer et Grey)	BHS110703.163	COI	BHS Mountain, Beijing	39.51	115.33
Sphingidae	*Marumba gaschkewitschi* (Bremer et Grey)	BHS110703.073	COI	BHS Mountain, Beijing	39.51	115.33
Sphingidae	*Marumba gaschkewitschi* (Bremer et Grey)	BHS110703.152	COI	BHS Mountain, Beijing	39.51	115.33
Sphingidae	*Marumba gaschkewitschi* (Bremer et Grey)	BHS110703.158	COI	BHS Mountain, Beijing	39.51	115.33
Sphingidae	*Marumba gaschkewitschi* (Bremer et Grey)	BHS110703.161	COI	BHS Mountain, Beijing	39.51	115.33
Sphingidae	*Marumba gaschkewitschi* (Bremer et Grey)	BHS110703.162	COI	BHS Mountain, Beijing	39.51	115.33
Sphingidae	*Marumba gaschkewitschi* (Bremer et Grey)	BHS110703.164	COI	BHS Mountain, Beijing	39.51	115.33
Sphingidae	*Marumba gaschkewitschi* (Bremer et Grey)	BHS110703.166	COI	BHS Mountain, Beijing	39.51	115.33
Sphingidae	*Marumba gaschkewitschi* (Bremer et Grey)	BHS110703.167	COI	BHS Mountain, Beijing	39.51	115.33
Sphingidae	*Phyllosphingia dissimilis* (Bremer)	BHS100703.013	COI	BHS Mountain, Beijing	39.51	115.33
Sphingidae	*Phyllosphingia dissimilis* (Bremer)	BHS100705.020	COI	BHS Mountain, Beijing	39.51	115.33
Sphingidae	*Phyllosphingia dissimilis* (Bremer)	BHS110703.157	COI	BHS Mountain, Beijing	39.51	115.33
Sphingidae	*Rhagastis mongoliana mongoliana* (Butler)	BHS100704.056	COI	BHS Mountain, Beijing	39.51	115.33
Sphingidae	*Rhagastis mongoliana mongoliana* (Butler)	BHS100705.010	COI	BHS Mountain, Beijing	39.51	115.33
Sphingidae	*Sphinx ligustri constricta* (Butler)	BHS100704.030	COI	BHS Mountain, Beijing	39.51	115.33
Sphingidae	*Sphinx ligustri constricta* (Butler)	BHS100705.018	COI	BHS Mountain, Beijing	39.51	115.33
Thyatiridae	*Tethea albicostata* (Bremer)	BHS100704.147	COI	BHS Mountain, Beijing	39.51	115.33
Thyatiridae	*Tethea albicostata* (Bremer)	BHS100703.144	COI	BHS Mountain, Beijing	39.51	115.33

Lepidostomat *Lepidostoma sp*	LS100909.174	COI	Lhasa, Tibet	29.3	91.15
Lepidostomat *Lepidostoma sp*	LS100909.176	COI	Lhasa, Tibet	29.3	91.15
Lepidostomat *Lepidostoma sp*	LS100909.183	COI	Lhasa, Tibet	29.3	91.15
